# GIPS-Mix for Accurate Identification of Isomeric Components in Glycan Mixtures Using Intelligent Group-Opting Strategy

**DOI:** 10.1021/acs.analchem.2c02978

**Published:** 2022-12-22

**Authors:** Chuncui Huang, Meijie Hou, Jingyu Yan, Hui Wang, Yu Wang, Cuiyan Cao, Yaojun Wang, Huanyu Gao, Xinyue Ma, Yi Zheng, Dongbo Bu, Wengang Chai, Yan Li, Shiwei Sun

**Affiliations:** † Institute of Biophysics, Chinese Academy of Sciences, 15 Datun Road, Beijing100101, China; ‡ Key Laboratory of Intelligent Information Processing, Institute of Computing Technology, Chinese Academy of Sciences, 6 Kexueyuan South Road, Beijing100080, China; § Dalian Institute of Chemical Physics, Chinese Academy of Sciences, Key Laboratory of Separation Science for Analytical Chemistry, Dalian116023, China; ∥ University of Chinese Academy of Sciences, 19 Yuquan Road, Beijing100049, China; ⊥ College of Information and Electrical Engineering, China Agricultural University, Beijing100083, China; # Glycosciences Laboratory, Department of Medicine, Imperial College London, LondonW12 0NN, United Kingdom

## Abstract

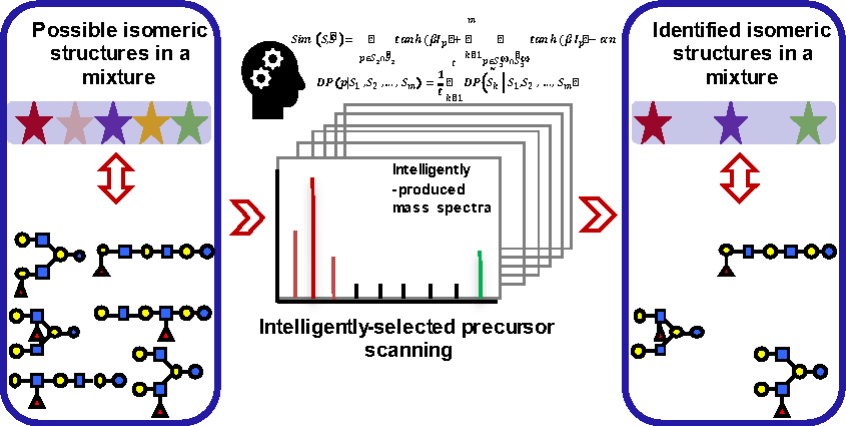

Accurate identification of glycan structures is highly desirable as they are intimately linked to their different functions. However, glycan samples generally exist as mixtures with multiple isomeric structures, making assignment of individual glycan components very challenging, even with the aid of multistage mass spectrometry (MS^n^). Here, we present an approach, GIPS-mix, for assignment of isomeric glycans within a mixture using an intelligent group-opting strategy. Our approach enumerates all possible combinations (groupings) of candidate glycans and opts in the best-matched glycan group(s) based on the similarity between the simulated spectra of each glycan group and the acquired experimental spectra of the mixture. In the case that a single group could not be elected, a tie break is performed by additional MS^n^ scanning using intelligently selected precursors. With 11 standard mixtures and 6 human milk oligosaccharide fractions, we demonstrate the application of GIPS-mix in assignment of individual glycans in mixtures with high accuracy and efficiency.

## Introduction

Glycans play important roles in a wide range of biological processes such as protein conformation, molecular recognition, cell proliferation and differentiation, and antiviral activity. These diverse functions of glycans are conferred by their different fine structures, and alternations of glycan structures can be associated with various diseases and cancers.^1,2^ Thus, understanding and defining the detailed structures of glycans as discrete components or in mixtures are extremely important.

Unlike the linear chain of proteins, branching is a unique feature of glycan structures. Increased branching of *N-*glycans has been shown to be related with invasiveness and metastatic potential of tumors.^3^ For example, an elevated level of *N-*glycans with bisecting *N-*acetylglucosamine (GlcNAc) occurs frequently in cancer cells, whereas isomeric triantennary branching is usually present in normal cells.^4−6^ Previous studies have indicated that multiple isomeric glycans including different epitopes exist as mixtures exclusively in biological samples.^7,8^ Unlike a pure glycan consisting of only a single component, a glycan mixture may contain a group of glycan components with different molecular masses or with identical masses as isomeric structures. Isomeric glycan structures, with either different branching patterns or different linkages, exist generally in glycoproteins, glycolipids, and secreted human milk.

Mass spectrometry (MS) has become the primary method for glycomic analysis due to its high sensitivity and the wealth of structural information obtainable from the comprehensive fragmentation. Primary MS and MS^2^ scanning are not always sufficient to provide detailed isomeric structural information on glycans due to the diverse branching patterns or linkages. Multistage MS (MS^n^) for sequential fragmentation has been successfully applied to determine glycan branching patterns of purified discrete glycans with^9,10^ or without^11^ computer-assisted program. It has also been reported in previous studies that isomeric glycans are always frequently sequenced together, thus producing mixed spectra containing fragment ions from multiple isomeric precursors.^7,8^ The complicated fragments and the diversity of glycan forms further increased the difficulty for identification, making it extremely challenging to analyze glycans with isomeric branching patterns within a mixture.

Glycan profiling by MALDI-MS is a widely used approach for rapid analysis of glycan mixtures released from glycoproteins and glycolipids from cell and tissues. This approach has been applied to obtain primary information on glycan components within mixtures, e.g. estimation of glycan identities and their relative abundances.^12,13^ However, in most cases of glycan profiling, isomeric glycan components with identical masses are presented in an ambiguous form, e.g. structural cartoons with all possible branches listed in brackets. Detailed assignment of the glycans may have to rely on knowledges of biosynthetic pathways and individual’s research experiences if insufficient structural information can be obtained experimentally. Therefore, isomeric glycans in a mixture such as those with different epitopes and unique branches related to diseases are difficult to identify. In the previous attempts for identification of isomeric Lewis (Le) epitopes, e.g. Le^a^/Le^x^ or Le^b^/Le^y^, in mixtures, diagnostic ions and the relative intensity ratios of specific fragments were used to match the sample to a spectrum library of known epitopes, but the identification has to rely on diagnostic fragments and existing spectrum library, and experts’ experience as well.^8^ Also, these empirical and manual assignments of mixed structures were confined to well-defined mixtures containing limited numbers of residues, and can only be achieved in specialized laboratories.^7,14^

Major efforts have been made to the development of computation approaches to assist in glycan structure determination by either analysis of the large MS data acquired^15^ or guidance to product-ion scanning,^9^ but most of these approaches were designed to assign a glycan in a relatively pure state.^9−11,15^ Recently, a modular glycan mapping method, StrucGP, has been developed to distinguish different isoforms.^16^ The authors also stated that the approach can only identify one isomer per spectrum and thus cannot be used to deduce isomeric glycan components from the spectrum of a glycan mixture.^16^ In our previous studies, the concept and approach of glycan intelligent peak selection (GIPS) in conjunction of MALDI-MS^n^ were proposed to identify branching patterns of individual glycans purified by chromatographic means.^9,10^ The application of these methods targeting discrete glycans may be limited as it requires prior multiple chromatographic fractionation which can be laborious and difficult. Detailed analysis of glycan components including complex isomeric glycans in mixtures is highly desirable.

We now develop further the GIPS-based computational MS^n^ approach, GIPS-mix, to attempt mixture analysis for glycan isomers. Using 11 standard binary mixtures made from purified oligosaccharides with different molar ratios and six naturally derived glycan mixtures, human milk oligosaccharide (HMO) mixtures with various degrees of polymerization (DP), as representatives, we demonstrate that the GIPS-mix approach in combination with MALDI-MS^n^ scanning can be successfully used to assign isomeric branching patterns of glycan components in mixtures with high accuracy and efficiency. Although presentative samples in the present work were all successfully resolved using MS^2^ and MS^3^ scanning, MS^n^ is required to handle more complex sequences, such as the purified *N-*glycans we demonstrated previously.^10^

## Methods

### Materials

Glycan standards were purchased from Dextra Laboratories (Reading, England) and Elicityl (Crolles, France). Sodium chloride, acetic acid, sodium hydroxide, and standard peptides were from Sigma-Aldrich (St. Louis, MO). Acetonitrile, ethanol, and water were obtained from Avantor Performance Materials (Center Valley, PA). C18-Sep-Pak cartridge was obtained from Waters (Milford, MA).

### HPLC Fractionation and ESI-CID-MS/MS Analysis of Neutral Human Milk Oligosaccharides

The extraction and fractionation of HMOs were performed as previously reported and neutral fraction DP4 to DP10 were obtained.^17^ The DP7 fraction was further separated by PGC columns (Hypercarb, 5 μm, Thermo Fisher Scientific, Waltham, MA) to obtain isomeric heptasaccharides by acetonitrile/water gradient elution at a flow rate of 0.8 mL/min for preparative (4.6 × 150 mm) and 0.2 mL/min for analytical scale (2.1 × 100 mm) separation.

Negative-ion ESI-CID-MS/MS of the purified DP7 subfractions was carried out on a SCIEX X500B instrument (AB Sciex). The ion source gas was at 55 psi and temperature at 550 °C. The desolvation and capillary voltage was 70 and 4500 V, respectively, and the collision energy was 50 V.

### ^1^H NMR of Human Milk Nonasaccharides

^1^H NMR spectra were recorded at 600 MHz on an Agilent DD2 instrument equipped with a cryoprobe probe, in D_2_O at 25 °C. The observed ^1^H chemical shifts were relative to internal acetone (2.225 ppm). Each sample (∼500 μg) was deutrium exchanged twice with D_2_O 99.9% (Cambridge Isotope Laboratories, MA) before being dissolved in 550 μL of D_2_O and transferred to a conventional 5 mm NMR tube for analysis.

### MALDI-MS of Permethylated Glycans for GIPS-Mix

Permethylation and purification were performed as previously reported using the NaOH slurry method.^18^ The permethylated sample in the chloroform layer was purified on a C18 Sep-Pak cartridge.

The permethylated samples were dissolved in methanol and applied to a μfocus MALDI plate target (Hudson Surface Technology, NJ) before addition of a matrix solution (0.5 μL) of 2,5-dihydroxybenzoic acid (20 mg/mL) methanol/water (1:1) containing 0.1% trifluoroacetic acid and 1 mM NaCl. The analysis was carried out on a Shimadzu Axima Resonance instrument.

For mixture analysis by GIPS-mix, primary MS and MS^2^ production-ion spectra were initially acquired. The fragment ions in the MS^2^ spectrum with relative intensities above 10% were selected as precursors for MS^3^ scanning, and all MS^2^ and MS^3^ spectra were fed into GIPS-mix program as mzXML files for initial analysis by the first module, the*“glycan grouping and opting”* module of the GIPS-mix. If a single solution (i.e., one *candidate group of isomeric glycans*, see below for description) is obtained, the process terminates. Otherwise, further analysis by the second module, the “*tie breaking”* module, is carried out until a single solution is reached.

### GIPS-Mix Approach—The “Glycan Grouping and Opting” Module

This module enumerates all possible groupings of the isomeric candidate glycans in the mixture and simulates the glycosidic cleavages to construct the theoretical mass spectra of each candidate group, and finally selects the best-matched glycan group(s) based on the similarity between theoretical spectra of each candidate group and experimental spectra of the mixture. The key steps of the module are described as below.

i *Generating isomeric candidate glycans in the mixture:* For a glycan mixture, we first produce its MS^n^ spectra. These experimental MS^n^ spectra are denoted as *S* = {*S*^1^, *S*^2^, *S*_1_^3^,···, *S*_*n*_^3^}, where *S*^1^, *S*^2^ represent the MS^1^, MS^2^ spectrum, respectively, and *S*_*i*_^3^ represents a MS^3^ spectrum produced by using a selected fragment ion in the MS^2^ spectrum as the precursor. All the spectra generated are used to form a tree (called *spectra tree*).
  Based on MS^1^ spectrum *S*^1^, GIPS-mix determines the molecular mass of the isomeric glycans in the mixture. All glycans in the glycan sequence database (GlyTouCan^19^ used in the study) with this molecular mass are listed as candidate glycans.
ii *Glycan grouping:* The isomeric glycan components in the mixture can be considered as a group of candidate glycans. To identify these glycan components, we enumerate all possible groupings of the candidate glycans and denote them as candidate groups. Specifically, suppose that we have already identified *n* candidate glycans *g*_1_, *g*_2_,..., *g*_*n*_ as described in the previous step, we will have a total of 2^*n*^ – 1 possible groupings of these glycans, i.e., {*g*_1_}, {*g*_2_},..., {*g*_1_, *g*_2_,..., *g*_*n*_}. Each grouping represents a possible case of the actual isomeric glycan components within the mixture, and the remaining task is to identify the most likely candidate group.
iii *Predicting theoretical spectra for each candidate group:*
  The theoretical spectrum of a glycan group is defined as the union of theoretical spectra of all candidate glycans in the group. For a candidate group *G*_*i*_, we first generate its theoretical MS^2^ and MS^3^ spectra of each of its candidate glycans in *G*_*i*_ based on glycosidic fragmentations^20^ before construction of the theoretical spectra as below.

We take the union of the theoretical MS^2^ spectra obtained from all candidate glycans and use the union as the theoretical MS^2^ spectrum of the candidate group, i.e.,
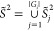


(: theoretical MS^2^ spectrum of candidate group *G*_*i*_; : theoretical MS^2^ spectrum of the j-th candidate glycan in *G*_*i*_).

### MS

For the k-th experimental ^3^ spectrum *S*_*k*_^3^, we construct a counterpart theoretical *MS*^3^ spectrum  as follows: from each candidate glycan in *G*_*i*_, we extract the fragments with identical mass to the precursor ion of *S*_*k*_^3^ and simulate cleavage of these fragments, yielding a collection of theoretical spectra (denoted as ) as the results. Similar to , the union of these theoretical spectra , forms the theoretical MS^3^ spectra of the candidate group *G*_*i*_.

As results, for each candidate group, we obtain its theoretical spectra  as counterparts of the experimental spectra .

iv *Evaluating the candidate groups:*
  As MS^2^ is not sufficient for analysis of mixtures containing isomeric glycans,^7,14^ GIPX-mix starts from MS^3^ combined with MS^2^. For each candidate group, we calculate the similarity between its theoretical  and experimental spectra *S* = {*S*^2^, *S*_1_^3^, *S*_2_^3^,···, *S*_*k*_^3^} of the mixture. The similarity measures the common peaks shared by the experimental spectra and their counterpart theoretical spectra as follows.

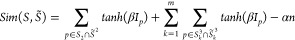
1

Here,  represents a peak shared by experimental MS^2^ spectrum *S*_2_ and its counterpart theoretical spectrum . Similarly,  represents the common fragment peaks shared by the experimental and the corresponding theoretical MS^3^ spectrum. Without additional hyperparameters referring to the stages of MS scanning, the weights of MS^2^ and MS^3^ spectrum are the same.

The term *I*_*p*_ represents the intensity of ion peak *p*. Although a higher intensity peak may be more useful in assignment of the glycan structure, the contribution or the weight of ion intensities to the assignment may not be reflected by the true scale of *I*_*p*_ intensity difference between the high and low intensity ions (e.g., the weight difference of 10 between ions with 80% and 8% relative intensities). To overcome this, we use *tanh*(β*Ip*) to reduce the actual scale of intensity difference as the range of *tanh* is only betwen [0, 1].

The term – α*n* (*n*, the number of glycan candidates within the group) is also introduced to handle the situation when multiple candidate groups are obtained without a sole solution. The introduction of – α*n* allows GIPS-mix to select the candidate group with least number of components. In the study, we tested different scaling parameters before the selection of α = 1 and β = 0.2 to allow for reliable identifications.

v *Opting in the best-matched candidate group(s):* We rank the candidate groups according to formula (*I*) and then select the top candidate group(s) with the highest value(s) as the final result. In the case that a single glycan group cannot be extracted from the multiple solutions calculated, further operations are performed, as described below, to break the tie.

### GIPS-Mix Approach—The “Tie Breaking” Module

The result of multiple best-matched groups indicates that the acquired experimental spectra cannot provide sufficient information to distinguish these groups. To break the tie among these glycan groups, we select a fragment ion, which is potentially most useful for tie-breaking, to generate a further product-ion spectrum. For selection of this fragment, we used the “*distinguishing power value* (*DPv*)” concept described previously^9^ and calculate the *DPv* of each fragment ion in the experimental spectrum. The fragment with the highest *DPv* is selected as the precursor-ion for a further round of product-ion scanning. The spectrum thus obtained can be very useful for differentiation of these candidate groups. The calculation of *DPv* is based on the decrease of probability entropy of all the candidate groups calculated as described previously for a single purified component.^9^
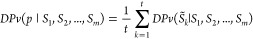


Here, *DP*(*p*|*S*_1_, *S*_2_,···, *S*_*m*_) represents the expected information gain when using *p* as precursor-ion.

The probability of each candidate group *G*_*i*_ to be the actual group is also calculated using the same empirical scoring function described previously^9^ for a single glycan component.
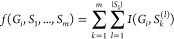


Here,  if *S*_*k*_^(*l*)^, the *l*-*th* peak in *S*_*k*_, can be explained by any candidate glycan in group *G*_*i*_ and , otherwise. For the purified individual glycans, the term *G*_*i*_ is an individual glycan, and here for mixture analysis, whereas here for mixture analysis, the *G*_*i*_ is all the candidate glycans in the group.

GIPS-mix uses the function *f*(*G*_*i*_, *S*_1_, *S*_2_,···, *S*_*m*_) to calculate the probability for each candidate group *G*_*i*_ likely to be the actual group. If a single glycan group has its probability higher than the predefined threshold, GIPS-mix terminates and reports the glycans in this group as the components of the mixture. Otherwise, GIPS-mix repeats the spectrum generating process until a single glycan group can be opted in with sufficiently high probability.

## Results and Discussion

### The Concept of GIPS-Mix

GIPS-mix aims to assign the isomeric glycan components within a mixture based on the MS^n^ spectra of the mixture. All the possible glycans with the identical molecular mass identified by MS^1^ are extracted from a glycan database (GlyTouCan^19^ used in the present study) and listed as *candidate glycans*. GIPS-mix then constructs hypothetically *candidate groups*, which are all the possible mixtures with different numbers and different combinations of components (the *candidate glycans*). GIPS-mix assigns the isomeric glycans by comparing the acquired MS^n^ spectra of the actual mixture with the computer simulated MS^n^ spectra of all hypothetical mixtures (the *candidate groups*).

Two computation modules were designed, the *“glycan grouping and opting”* module and the “*tie breaking”* module (see Methods for details). The glycan grouping and opting module calculates the similarity between the theoretical spectra of each candidate group and experimental spectra of the actual mixture, and opts in the best-matched candidate group(s). By comparing the theoretical spectra of candidate group rather than those of individual glycans, GIPS-mix can explore more thoroughly the information contained in the experimental spectra of the mixture (Figure S1).

In the case that a single solution could not be reached, *tie breaking* will be performed to distinguish the remaining candidate groups. In this module, further MS scanning is required and the selection of suitable precursor is guided by the *DPv* (see Methods for detail) until a single solution with only one candidate group is identified.

### Identification of Glycan Components in Standard Mixtures

**Figure 1:**
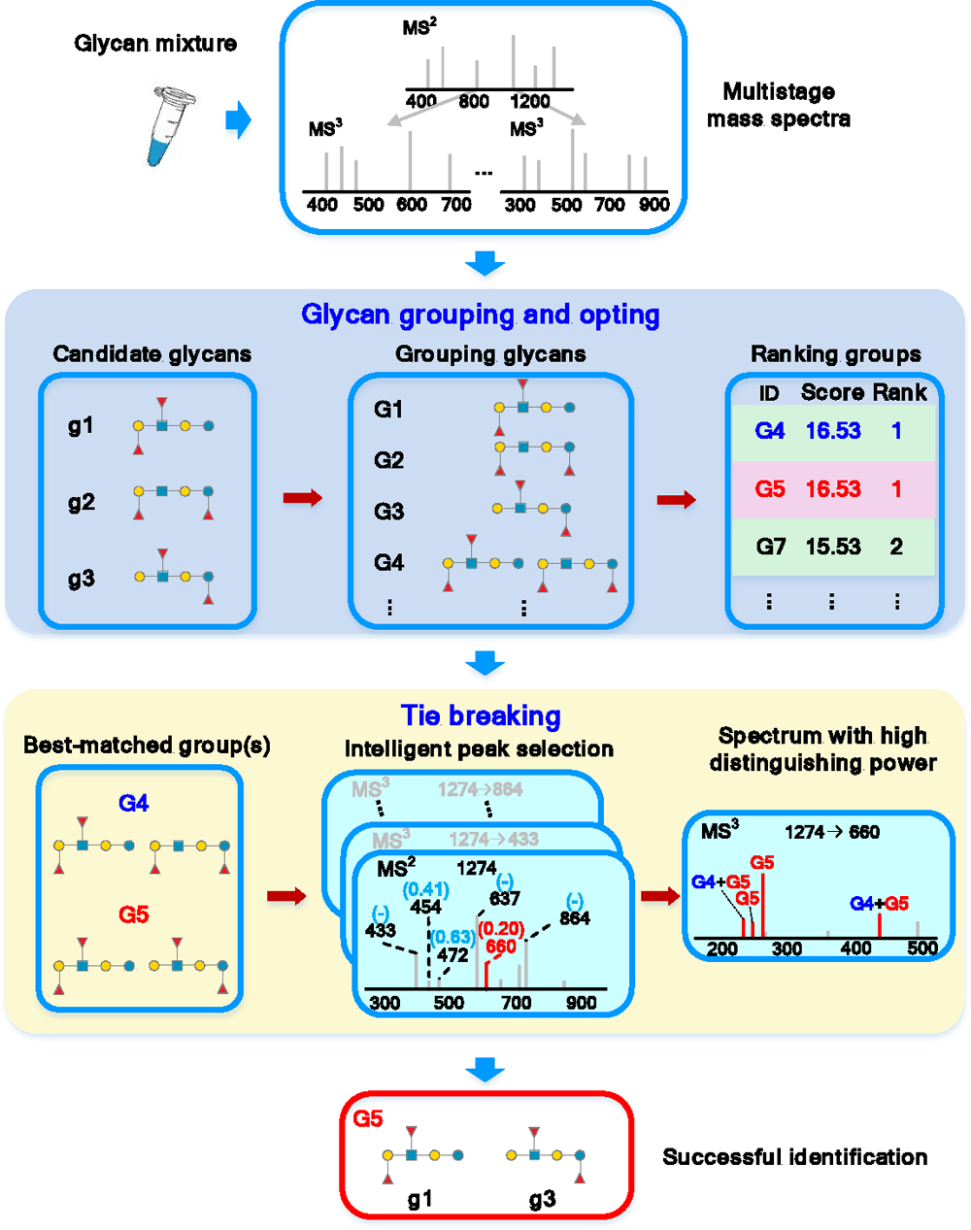
Identification process of a sample mixture, LNDFH-I and LNnDFH-II with a molar ratio 1:1, using GIPS-mix. The MS^1^ spectrum gave a single MNa^+^ at *m*/*z* 1274 and 8 candidate glycans with 3 different branching patterns were selected from the database GlyTouCan. For each branching pattern, one glycan was used as the representative, denoted as *g*_1_, *g*_2_, *g*_3_, respectively (*g*_1_ denotes LNDFH-I, and *g*_3_ denotes LNnDFH-II). The *glycan grouping and opting* module enumerated 7 groupings. Among these groups, *G*_4_ and *G*_5_ showed the highest similarity (16.53) between their theoretical and the experimental spectra. To distinguish these two groups, the *tie breaking* module selected *m*/*z* 660 with the highest distinguishing power (0.20) as the precursor for MS^3^ scanning. Based on MS^3^ spectrum together with the spectra already-acquired, GIPS-mix assigned *G*_5_ = (*g*_1_, *g*_3_), i.e., LNDFH-I and LNnDFH-II, as the components of the mixture.

**Figure 2:**
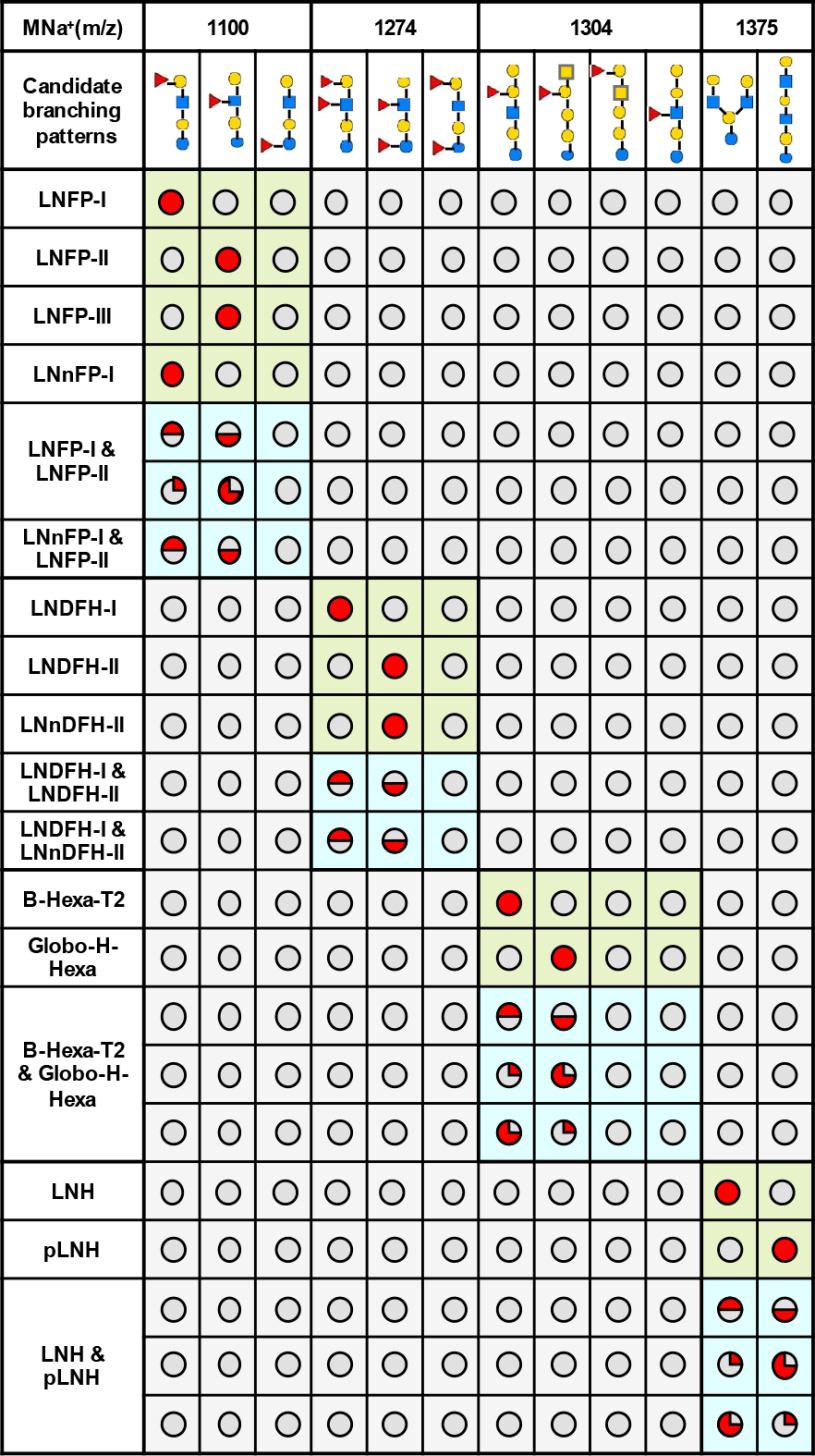
Identification of components in standard mixtures and discrete glycans by GIPS-mix. The compositions of standards are showed by the circles with red/gray colors, and all the mixtures and discrete glycans were identified successfully.

Using a mixture of LNDFH-I and LNnDFH-II, with a molar ratio of 1:1, as an example (Figure 1 and Figure 2), we describe the main steps of the GIPS-mix approach for identification of isomeric glycans in a mixture and how the process is accomplished in an interactive manner with the mass spectrometer rather than using preprogrammed parameters.

MS^1^ of the glycan mixture gave a permethylated MNa^+^ at *m*/*z* 1274 (Figure 1), indicating the glycans with a molecular mass of 999. All eight glycans with this mass were considered as candidate glycans and extracted from the database. These can be classified as 3 different branching patterns (number of branching patterns, NoB = 3) listed as *g*_*1*_ to *g*_*3*_ (Figure 1 and Table S1). The possible number of candidate groups (NoC) were calculated, and 7 possible combinations of the candidate glycans were obtained (NoC = 7, Table S1). The MS^2^ spectrum from the precursor *m*/*z* 1274 was then produced. Fragment ions with relative intensity >10% were further fragmented by MS^3^ scanning (Figure S2a–f). All the resulting fragment ion peaks were overlaid together to form an overall spectrum, the spectra-tree. Theoretical MS^2^ and MS^3^ spectra of all the candidate glycans in each group were simulated by considering single and double glycosidic cleavage only. A *glycan grouping and opting* module with a score function (see Methods for details) was utilized to determine the candidate groups which can best explain the experimental spectra. For candidate group {*g*_*1*_, *g*_*2*_, *g*_*3*_}, the similarity value between its theoretical, and the experimental spectra was calculated to be 15.53; for candidate group {*g*_*1*_, *g*_*2*_} and {*g*_*1*_, *g*_*3*_}, the similarity was 16.53. Therefore, {*g*_*1*_, *g*_*2*_} and {*g*_*1*_, *g*_*3*_} were calculated to be the best results.

Distinction of these two best-fit candidate groups was then made by the *tie breaking* module. In this module, the *DPv*s of all the fragments in the acquired MS^n^ spectra were calculated (see Methods for detail). Among the major ions in the MS^2^ spectrum (Figure 1), the *DPv*s of *m*/*z* 454 and 472 were the highest. However, the product-ion spectra from these two precursors did not give a definitive solution, probably due to their low intensities and unuseful fragmentation. In this case, peak *m*/*z* 660 with the next highest *DPv* (0.20) was selected as the precursor for MS^3^, and finally glycans in the candidate group {*g*_*1*_, *g*_*3*_} were identified to be the actual components.

In the MS^3^ spectrum of *m*/*z* 660 (Figure S2g), the fragments *m*/*z* 241 and 259 were unique for the candidate glycan *g*_*3*_, and therefore among the two possible groups {*g*_*1*_, *g*_*2*_} and {*g*_*1*_, *g*_*3*_}, the candidate group {*g*_*1*_, *g*_*3*_} was reported as the glycan components in the mixture due to the identification of glycan *g*_*3*_.

The key to success in the *tie breaking* module is the finding and use of the distinctive fragments, e.g. in this example *m*/*z* 660/472/259 for Le^x^ epitope as *g*_*3*_ (Table S2). Further examples of the distinctive fragments identified by GIPS-mix which can be used for identification of candidate groups include *m*/*z* 690/660/433 for LNFP-I carrying the blood-group H epitope, *m*/*z* 660/442/259 for LNFP-II carrying the Le^a^ epitope, and *m*/*z* 637/449/431 for B-Tetra-T2 (Table S2). These distinctive fragments determined by GIPS-mix and used for differentiation of mixtures are in accordance with the previous reported ions generated experimentally (Table S2).

Using GIPS-mix, we successfully identified mixtures of standard glycans with different ratios (Figure 2 and Table S1), including LNFP-I and LNFP-II (1:1, 1:3), LNFP-II and LNnFP-I, LNH and pLNH (1:1, 1:3, 3:1), and B-Tetra-T2 and Globo-H-Hexa (1:1, 1:3, 3:1) (Figures S3–S12), and 11 discrete HMOs (Figure 2 and Table S3). For a glycan sample isolated from a biological source, GIPS-mix can be used to identify the branching patterns of all the glycan components. In the case of only one glycan present in the sample, the *glycan grouping and opting* module is able to identify this situation (*g*_*i*_ = *g*_*1*_) and assign the branching pattern of the single glycan component (*g*_*1*_).

### Application of GIPS-Mix to Assignment of Individual Components in HMO Fractions

GIPS-mix was then applied to analyze neutral HMO fractions DP4 to DP9, obtained from HPLC.

**Figure 3:**
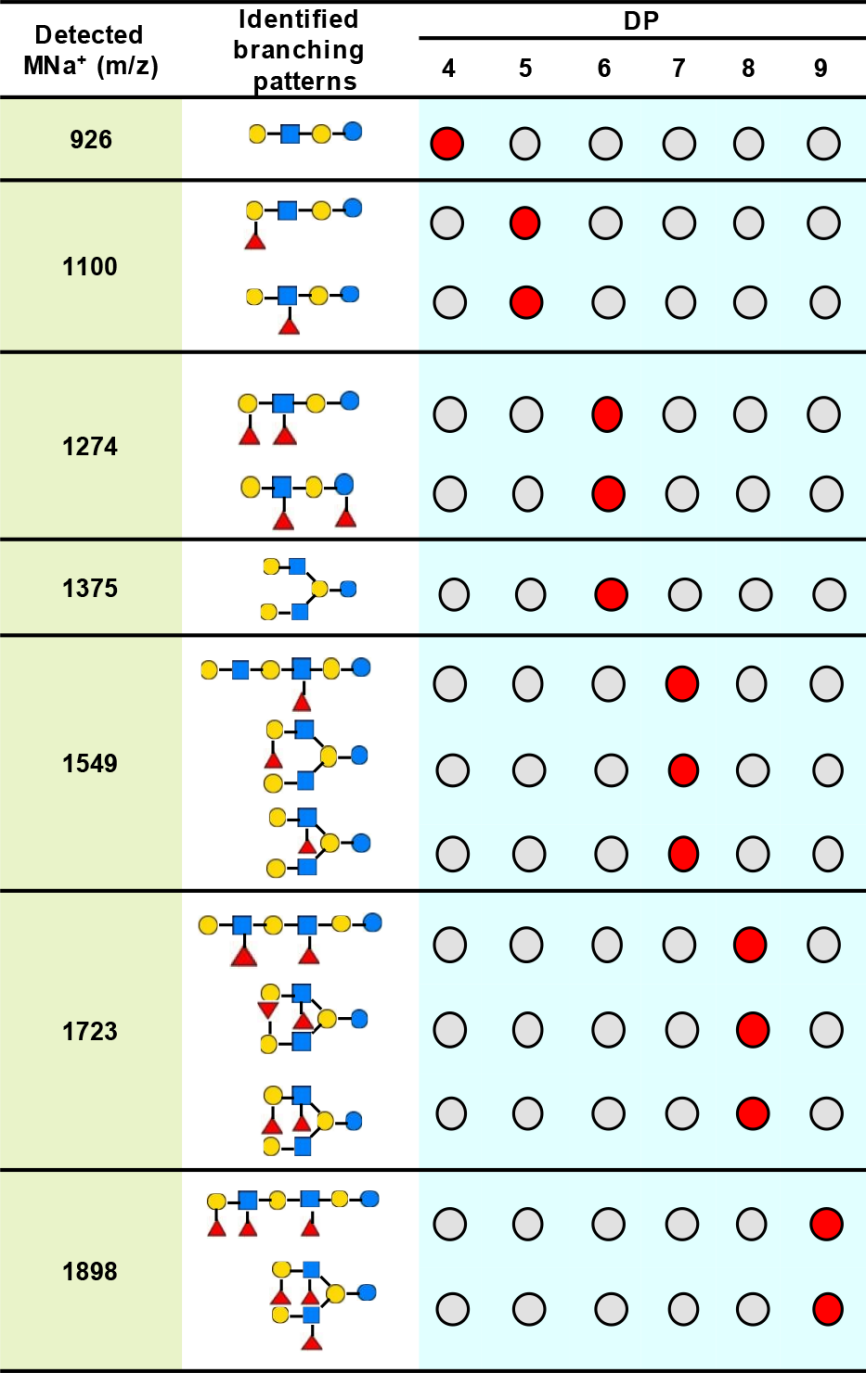
Assignment of components in neutral HMO mixture of DP4–DP9 by GIPS-mix.

In fraction DP4, a single MNa^+^ at *m*/*z* 926 with a composition of Hex3.HexNAc1 was detected. Only a linear sequence (Figure 3) was identified, in agreement with a previous report.^21^ In fraction DP5, two different branching patterns, a linear blood group H-containing and a branched Le^a/x^-containing sequence, both with MNa^+^ at *m*/*z* 1100, were identified as previously reported.^21−23^

In fraction DP6, two molecular species were detected inMS^1^: MNa^+^ at *m*/*z* 1274 (Fuc2.Hex3.HexNAc1) and *m*/*z* 1375 (Hex4.HexNAc2) (Figure 3). For the difucosylated hexasaccharide *m*/*z* 1274, two different branching patterns were identified. One of the Fuc residues is at the GlcNAc, while the other is at either the nonreducing terminal Gal forming a Le^b/y^ or the reducing terminal Glc forming a Le^a/x^ epitope (Figure 3).^21,23^ The nonfucosylated hexasaccharide *m*/*z* 1375 was identified as a branched hexasaccharide backbone sequence (Figure 3).^11,21,23,24^

Three different structures with MNa^+^*m*/*z* 1549 were identified in fraction DP7, and these include monofucosylated linear and branched hexasaccharide backbones (Figure 3). For the two branched sequences the Fuc residue is either on the terminal Gal or an internal GlcNAc, forming a blood group H and Le^a/x^ antigen, respectively.^11,21,24,25^

For the difucosylated hexasaccharide fraction DP8, again one linear and two branched backbone sequences were identified with MNa^+^*m*/*z* 1723 (Figure 3). For the linear backbone, both Fuc residues were at the GlcNAc residues comprising a terminal and an internal Le^a/x^ epitope. For the two branched sequences, the two Fuc residues were either on the same branch to constitute a Le^b/y^ or at different branches forming a H or Le^a/x^ antigens.^21,26,27^

A linear and a branched backbone sequences with a MNa^+^*m*/*z* 1898 were found in the fraction of DP9 (Figure 3). For the linear backbone sequence, the three Fuc residues constituted a Le^b/y^ and an internal Le^a/x^ epitope, while for the branched sequence, the Le^b/y^ and Le^a/x^ epitopes are on the different branches as the reported TFLNH and TFpLNH structures.^21,28,29^

### Validation of the GIPS-Mix Assignments of Individual Components in the HMO Fractions

The branching patterns identified in the HMO fractions by GIPS-mix were then validated using DP7 and DP9 as the examples by the well-established methods, negative-ion -ESI-CID-MS/MS^30,31^ and NMR spectroscopy.^32^

**Figure 4:**
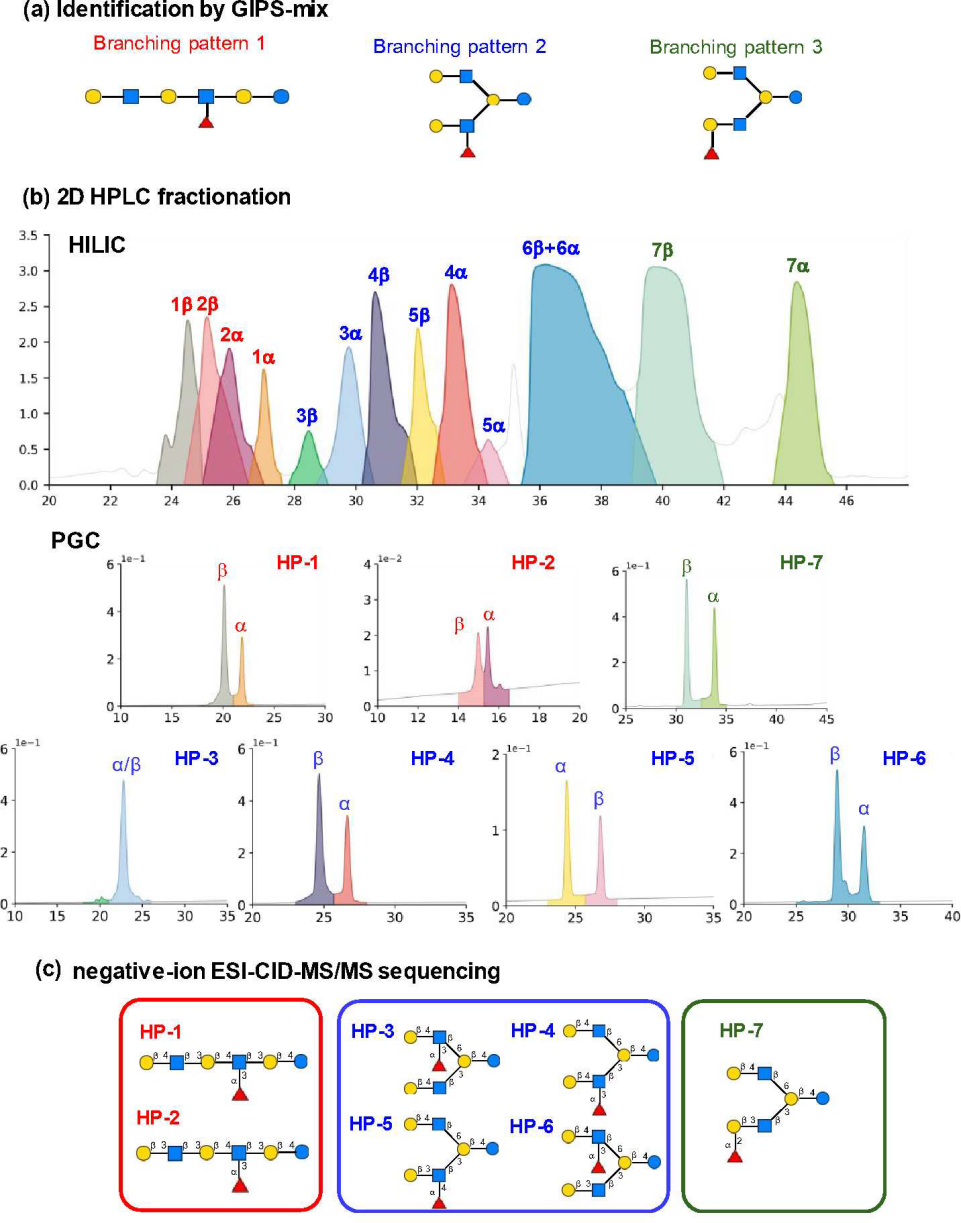
Identification of individual components in the fraction of DP7 after PGC-HPLC separation.The branching patterns identified in HMO fraction DP7 by GIPS-mix (a) were corroborated by negative-ion ESI-CID-MS/MS (c) after PGC-HPLC separation (b).

For DP7, incomplete HILIC fractionation gave a very complex profile (Figure 4b) and seven components, each with α and β anomers, were collected and further purified by repeated PGC-HPLC (Figure 4b). The sequences of the seven components were assigned by negative-ion ESI-CID-MS/MS^30−32^ as MFpLnNH, MFpLNH, MFLNnH-II, MFLNnH-I, MFLNH-II, MFLNH-III, and MFLNH-I^11,21,24,25^ (Figure 4c), in agreement with the three branching patterns determined by GIPS-mix (Figure 4a).

**Figure 5:**
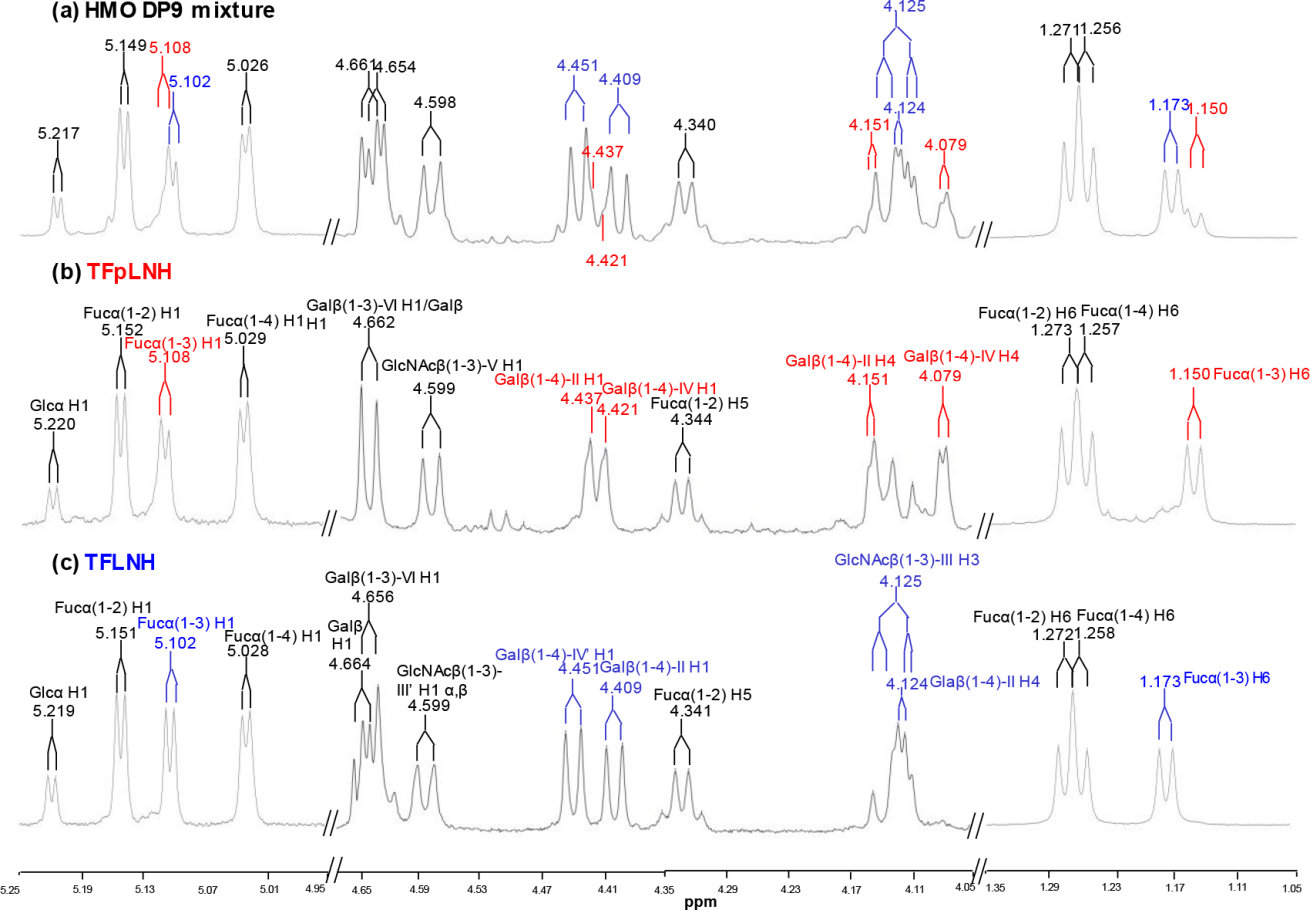
^1^H NMR spectra of HMO DP9 and standard nonasaccharides. HMO fraction DP9 (a) is compared with the spectra of two human milk nonasaccharides: linear TFpLNH (b) and branched TFLNH (c). Chemical shifts of characteristic peaks from TFpLNH are in red and those from TFLNH in blue. The chemical shifts of common peaks are in black.

^1^H NMR was used for validation of the results obtained for fraction DP9. From the proton spectrum (Figure S13a), two main components, TFpLNH and TFLNH, could be identified based on the key proton signals compared with literature data.^27,28^ To assist the chemical shift assignment, spectra of individual TFpLNH and TFLNH standards were also acquired (Figures S14 and S15), and the observed chemical shifts of the characteristic peaks (Tables S4 and S5) are identical to those of the respective ones published previously.^27,28^ The selected regions of the proton spectrum of DP9 (Figure 5a) can be compared with those of TFpLNH (Figure 5b) and TFLNH (Figure 5c). The major signals observed in the spectrum of DP9 (Figure 5a and Table S6), e.g. the signals of the subreducing terminal Gal at 4.437 and 4.409 ppm, are consistent with the presence of TFpLNH and TFLNH in the mixture. The presence of only two main components in the mixture were confirmed by subsequent PGC-HPLC analysis (Figure S13b).

## Conclusions

Branching pattern assignment of individual isomeric glycans in a mixture based on MS^n^ of all isomers was achieved by GIPS-mix. Integration of each candidate group into a virtual glycan was designed as an additional important element of the intelligent precursor selection strategy previously developed.^9^ The ability to identify branching patterns of isomeric glycan in a mixture was demonstrated by assignment of standard glycan mixtures and applied to analysis of complex HMO fractions. As for structural analysis of glycans using any conventional methods, the larger-sized glycans (e.g., DP > 20) are much more difficult than the smaller-sized ones (e.g., DP < 8), particularly in a mixture when the number of components is large.

In this proof-of-concept, we showed the initial success of GIPS-mix in branching pattern analysis based on glycosidic cleavage only, but isomeric sequence with different linkages was not considered. However, the concept, the basic idea, the algorithms, and the operation can be extended to assignment of monosaccharide linkages when double glycosidic cleavage and cross-ring fragmentation are used.^33^ Assignment of multiple isomeric structures with different linkage features within a mixture is undoubtedly more challenging but will be important for analysis of glycan mixtures obtained from biological sources without the need for prior separation into individual isomeric components, particularly in conjunction with glycan profiling.

## Data Availability

The GIPS-mix software is available through http://glycan.ict.ac.cn/GIPS. The MS^n^ spectra and computational process data are available in the Supporting Information.

## Supporting Information Available

Tables and figures illustrating identification process of standard glycan mixtures, supplemented illustration of the strategy, validation of identification results, and supplementary references (PDF)
MS raw data (XLSX)
Computational process data (XLSX)
