# Supplementary material for: GIPS-Mix for Accurate Identification of Isomeric Components in Glycan Mixtures Using Intelligent Group-Opting Strategy
Source: Anal Chem. 2022 Dec 22;95(2):811–9. doi: 10.1021/acs.analchem.2c02978 (PMC9850354; doi:10.1021/acs.analchem.2c02978)
Supplement: Supplementary file 1 — ac2c02978_si_001.pdf [file ac2c02978_si_001.pdf]

Supporting Information

GIPS-mix for accurate identification of isomeric components in glycan mixtures using intelligent group-opting strategy

Chuncui Huang[1†], Meijie Hou[2,4†], Jingyu Yan[3†], Hui Wang[2,4], Yu Wang[2,4], Cuiyan Cao[3], Yaojun Wang[5], Huanyu Gao[1], Xinyue Ma[1,4], Yi Zheng[3], Dongbo Bu[2,4], Wengang Chai[6*], Yan Li[1,4*], Shiwei Sun[2,4,*]

[1]Institute of Biophysics, Chinese Academy of Sciences, 15 Datun Road, Beijing 100101, China.

[2]Key Laboratory of Intelligent Information Processing, Institute of Computing Technology, Chinese Academy of Sciences, 6 Kexueyuan South Road, Beijing 100080, China.

[3]Dalian Institute of Chemical Physics, Chinese Academy of Sciences, Key Laboratory of Separation Science for Analytical Chemistry, Dalian 116023, China.

[4]University of Chinese Academy of Sciences, 19 Yuquan Road, Beijing 100049, China.

[5]College of Information and Electrical Engineering, China Agricultural University, 100083, China.

[6] Glycosciences Laboratory, Department of Medicine, Imperial College London, London W12 0NN, United Kingdom.

† C. H., M. H. and J. Y. contributed equally to this work.

*Correspondence authors. Email: w.chai@imperial.ac.uk (W. C.); yanli@ibp.ac.cn; (Y. L.); dwsun@ict.ac.cn (S. S.)

Table of Contents:

Table S1. Identification of components in standard HMO mixtures by GIPS-mix.

Table S2. Comparison of distinctive fragments calculated by GIPS-mix and reported in the literature.

Table S3. Identification of discrete HMOs by GIPS-mix.

Table S4. ^{1}H-NMR chemical shifts of the linear nonasaccharide TFpLNH.

Table S5. ^{1}H-NMR chemical shifts of branched nonasaccharide TFLNH.

Table S6. Comparison of ^{1}H-NMR chemical shifts of HMO fraction DP9 with TFpLNH and TFLNH.

Figure S1. The limitation of the individual-mixture comparison (IMC) strategy and the advantage of the group-mixture comparison (GMC) strategy used by GIPS-mix.

Figure S2. Produced MS^n spectra with peak annotation for the sample mixture, LNDFH-I and LNnDFH-II with a molar ratio 1:1, using GIPS-mix. (a), MS^2 spectrum of m/z 1274, (b) MS^3 spectrum m/z 433, (c) MS^3 spectrum m/z 449, (d) MS^3 spectrum m/z 637, (e) MS^3 spectrum m/z 834, (f) MS^3 spectrum m/z 864, (g) MS^3 spectrum m/z 660.

Figure S3. Identification by GIPS-mix of the components in a mixture of LNFP-I and LNFP-II with a molar ratio of 1:1.

Figure S4. Identification by GIPS-mix of the components in a mixture of LNFP-I and LNFP-II with a molar ratio of 1:3.

Figure S5. Identification by GIPS-mix of the components in a mixture of LNnFP-I and LNFP-II with a molar ratio of 1:1.

Figure S6. Identification by GIPS-mix of the components in a mixture of LNH and pLNH with a molar ratio of 1:1.

Figure S7. Identification by GIPS-mix of the components in a mixture of LNH and pLNH with a molar ratio of 1:3.

Figure S8. Identification by GIPS-mix of the components in a mixture of LNH and pLNH with a molar ratio of 3:1.

Figure S9. Identification by GIPS-mix of the components in a mixture of B-Tetra-T2 and Globo-H-Hexa with a molar ratio of 1:1.

Figure S10. Identification by GIPS-mix of the components in a mixture of B-Tetra-T2 and Globo-H-Hexa with a molar ratio of 1:3.

Figure S11. Identification by GIPS-mix of the components in a mixture of B-Tetra-T2 and Globo-H-Hexa with a molar ratio of 3:1.

Figure S12. Identification by GIPS-mix of the components in a mixture of LNDFH-I and LNDFH-II with a molar ratio of 1:1.

Figure S13. ^{1}H-NMR spectrum (a) and PGC-HPLC profile (b) of HMO fraction DP9.

Figure S14. ^{1}H-NMR spectrum of oligosaccharide TFpLNH.

Figure S15. ^{1}H-NMR spectrum of oligosaccharide TFLNH.

Supplementary References

Table S1. Identification of components in standard HMO mixtures by GIPS-mix

Sample	Mixed Components	MNa+ (*m/z*)	Ratio[1]	NoB[2]	NoC[3]	Assignment
**1**	LNFP-I & LNFP-II	1100	1:1	3	7	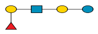 LNFP-I 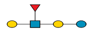 LNFP-II
**2**	LNFP-I & LNFP-II	1100	1:3	3	7	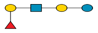 LNFP-I 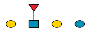 LNFP-II
**3**	LNnFP-I & LNFP-II	1100	1:1	3	7	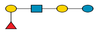 LNnFP-I 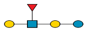 LNFP-II
**4**	LNH & pLNH	1375	1:1	2	3	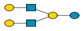 LNH pLNH
**5**	LNH & pLNH	1375	1:3	2	3	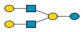 LNH pLNH
**6**	LNH & pLNH	1375	3:1	2	3	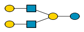 LNH pLNH
**7**	B-Hexa-T2 & Globo-H-Hexa	1304	1:1	4	15	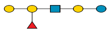 B-Hexa-T2 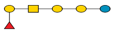 Globo-H-Hexa
**8**	B-Hexa-T2 & Globo-H-Hexa	1304	1:3	4	15	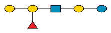 B-Hexa-T2 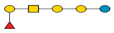 Globo-H-Hexa
**9**	B-Hexa-T2 & Globo-H-Hexa	1304	3:1	4	15	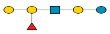 B-Hexa-T2 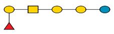 Globo-H-Hexa
**10**	LNDFH-I & LNDFH-II	1274	1:1	3	7	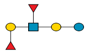 LNDFH-I 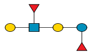 LNDFH-II
**11**	LNDFH-I & LNnDFH-II	1274	1:1	3	7	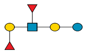 LNDFH-I 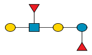 LNnDFH-II

[1]Molar ratio, [2]NoB: number of glycan branching patterns, [3]NoC: number of candidate groups.

Table S2. Comparison of distinctive fragments calculated by GIPS-mix and reported in the literature

Mixed Components	Epitopes	Distinctive fragment ions (m/z)	
		Identified by GIPS-mix	Supported by references*
LNFP-I & LNFP-II	H	690, 660, 433	690, 660, 433, 245, 229, 211 (Ref. S1, S2, S3)
	Le^{a}	660, 442, 259	864, 660, 586, 442, 259, 229 (Ref. S1, S2, S3)
LNnFP-I & LNFP-II	H	660, 415, 268	660, 472, 433, 415, 268, 245, 211 (Ref. S1, S2, S3)
	Le^{a}	660, 442, 259	864, 660, 586, 442, 259, 229 (Ref. S1, S2, S3)
B-Hexa-T2 & Globo-H-Hexa	B	637,449, 431	637, 449, 431, 419, 401, 259 (Ref. S1)
	H	660, 433, 415	912, 894, 660, 433, 415 (Ref. S1, S2, S3)
LNDFH-I & LNnDFH-II	Le^{b}	834, 433, 415	834, 760, 433, 415 (Ref. S1, S3)
	Le^{x}	660, 472, 259	660, 586, 472, 259, 229 (Ref. S1, S2, S3)
LNH & pLNH	LNH	486, 449, 259	486, 449, 440, 259 (Ref. S4)
	pLNH	935, 699, 472	935, 699, 486, 472 (Ref. S4, S5)

* The identical fragment ions are highlighted in red.

Table S3. Identification of discrete HMOs by GIPS-mix

Sample	Components	MNa+	NoB[1]	NoC[2]	Assignment
**1**	B-Hexa-T2	*m/z* 1304	4	15	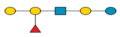 B-Hexa-T2
**2**	Globo-H-Hexa	*m/z* 1304	4	15	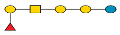 Globo-H-Hexa
**3**	LNDFH-I	*m/z* 1274	3	7	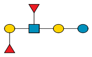 LNDFH-I
**4**	LNDFH-II	*m/z* 1274	3	7	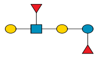 LNDFH-II
**5**	LNFP-I	*m/z* 1100	3	7	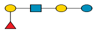 LNFP-I
**6**	LNFP-II	*m/z* 1100	3	7	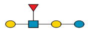 LNFP-II
**7**	LNFP-III	*m/z* 1100	3	7	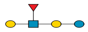 LNFP-III
**8**	LNH	*m/z* 1375	2	3	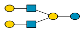 LNH
**9**	LNnDFH-II	*m/z* 1274	3	7	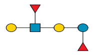 LNnDFH-II
**10**	LNnFP-I	*m/z* 1100	3	7	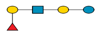 LNnFP-I
**11**	pLNH	*m/z* 1375	2	3	pLNH

[1]NoB: number of glycan branching patterns, [2] NoC: number of candidate groups.

Table S4. ^{1}H-NMR chemical shifts of the linear nonasaccharide TFpLNH.

```
                 Fucα1-4                Fucα1-3
                    |                      |
Fucα1-2Galβ1-3GlcNAcβ1-3Galβ1-4GlcNAcβ1-3Galβ1-4Glc
          VI        V          IV         III        II      I
```

Residue	H-1	H-2	H-3	H-4	H-5	H-6	NAc
Fucα(1-2)-	5.152				4.345	1.273	-
Galβ(1-3)-VI	4.662						-
Fucα(1-4)-	5.029				4.867	1.257	-
GlcNAcβ(1-3)-V	4.599		4.130				2.052
Galβ(1-4)-IV	**4.421**			**4.079**			-
Fucα(1-3)-	5.108				**4.812**	**1.150**	-
GlcNAcβ(1-3)-III	**4.725(α)** **4.711(β)**						**2.022**
Galβ(1-4)-II	**4.437**			**4.151**			-
Glcα	5.220						-
Glcβ	4.662	**3.278**				-	-

Chemical shifts in red are unique to TFpLNH, and chemical shifts in black are common to both TFpLNH and TFLNH.

Table S5. ^{1}H-NMR chemical shifts of branched nonasaccharide TFLNH.

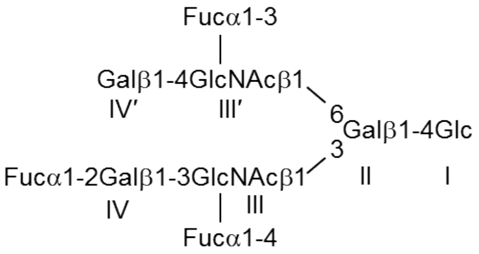

Residue	H-1	H-2	H-3	H-4	H-5	H-6	NAc
Fucα(1-2)-	5.151				4.341	1.272	-
Galβ(1-3)-IV	4.656			-	-	-	-
Fucα(1-4)-	5.028			-	4.865	1.258	-
GlcNAcβ(1-3)-III	4.608(α)<br>4.592(β)		4.125				**2.058**
Galβ(1-4)-IV'	**4.451**				-	-	-
Fucα(1-3)-	5.102			-	**4.821**	**1.173**	-
GlcNAcβ(1-6)-III'	**4.640**		-	-	-	-	2.050
Galβ(1-4)-II	**4.409**			**4.124**	-	-	-
Glcα	5.219			-	-	-	-
Glcβ	4.664	**3.288**		-	-	-	-

Chemical shifts in blue are unique to TFLNH, and chemical shifts in black are common to both TFLNH and TFpLNH.

Table S6. Comparison of ^{1}H-NMR chemical shifts of HMO fraction DP9 with TFpLNH and TFLNH.

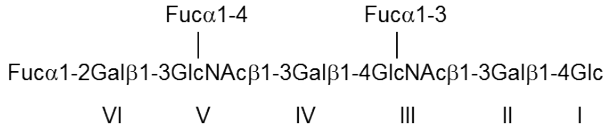

TFpLNH

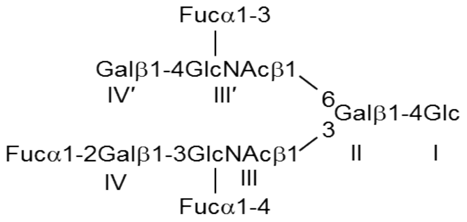

TFLNH

Residue	H-1	H-2	H-3	H-4	H-5	H-6	NAc
Fucα(1-2)-<br>Fucα(1-2)-	5.149				4.342	1.271	
Fucα(1-3)-<br>Fucα(1-3)-	5.108/5.102				4.812<br>4.821	1.150<br>1.173	
Fucα(1-4)-<br>Fucα(1-4)-	5.026				4.865	1.256	
-4Glcα<br>-4Glcα	5.217						
-4Glcβ<br>-4Glcβ	4.661						
-2Galβ(1-3)-VI	4.661	3.278<br>3.288					
-2Galβ(1-3)-IV	4.654						
-3,4GlcNAcβ(1-3)-V	4.598		4.125				2.052
-3,4GlcNAcβ(1-3)-III	4.608(α)<br>4.592(β)		4.125				2.058
-3Galβ(1-4)-IV	4.421			4.079			
Galβ(1-4)-IV	4.451			4.079			
-3,4GlcNAcβ(1-3)-III	4.724(α)<br>4.709(β)						2.022
-3,4GlcNAcβ(1-6)-III	4.634						2.052
-3Galβ(1-4)-II	4.437			4.151			
-3,6Galβ(1-4)-II	4.409			4.124			

Chemical shifts of characteristic peaks from TFpLNH are in red and those from TFLNH in blue. The chemical shifts of common peaks are in black.

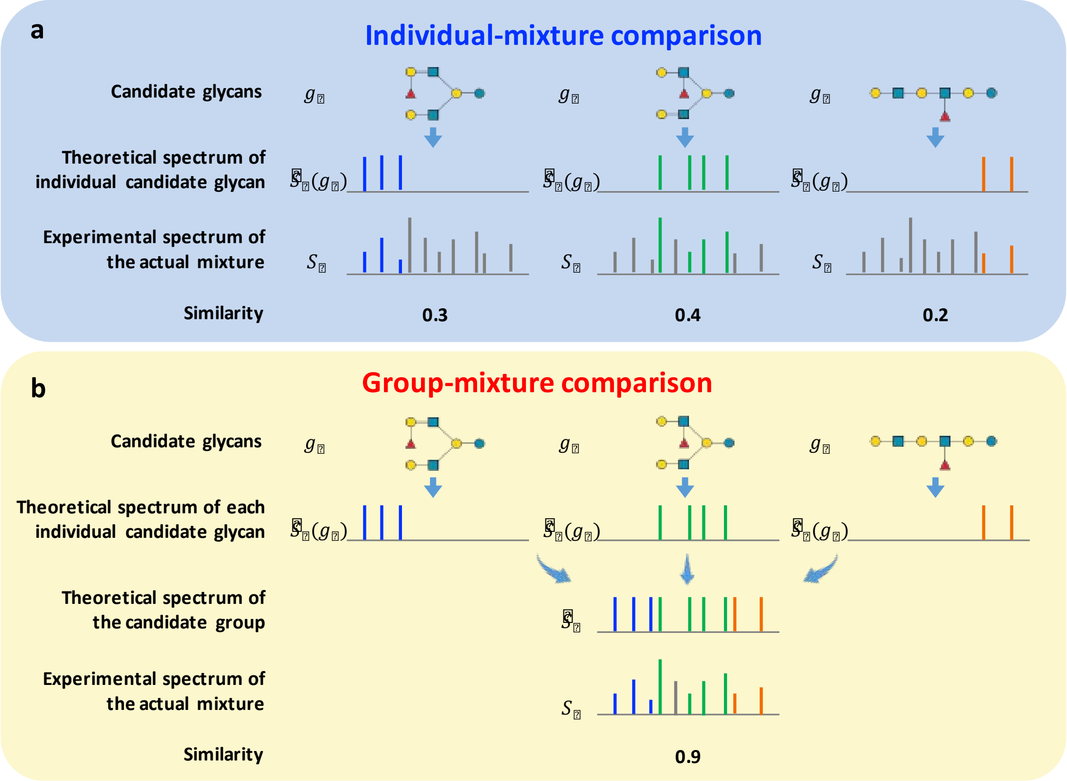

Figure S1. The limitation of the individual-mixture comparison (IMC) strategy and the advantage of the group-mixture comparison (GMC) strategy used by GIPS-mix.
(a) The IMC strategy compares theoretical spectrum of each individual candidate glycan with the experimental spectrum of the mixture sample. As the theoretical spectrum of an individual glycan is a subset of the experimental spectra of the mixture sample, the similarity between them is usually very low. In addition, it is also a great challenge to set a threshold of the similarity for reliable identification. **(b)** Unlike the IMC strategy, the GMC strategy first enumerates all possible groupings of the candidate glycans, constructs theoretical spectrum of each candidate group and compares it with experimental spectrum of the actual mixture. This way, it can fully exploit the information carried by the experimental spectra.

(a)

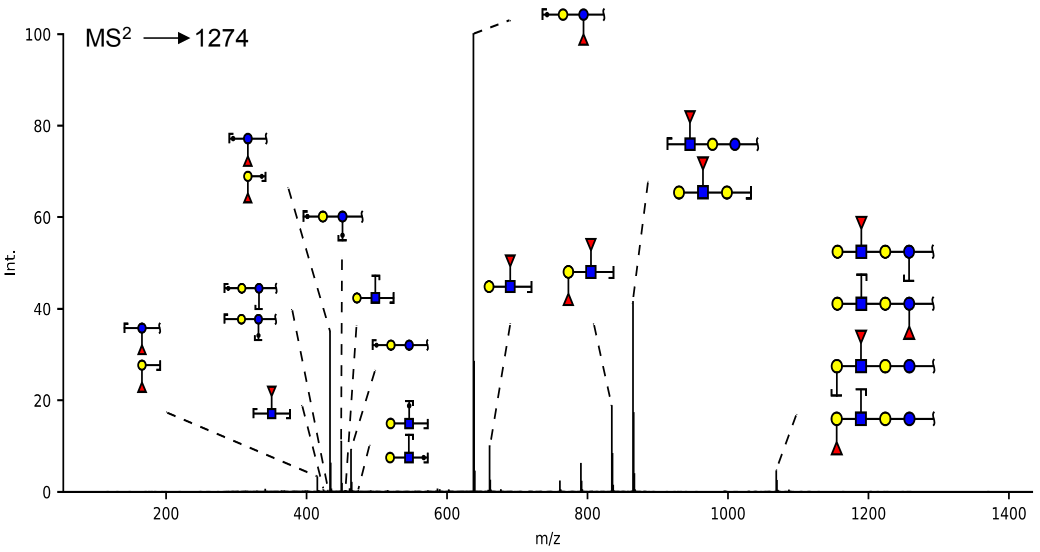

(b)

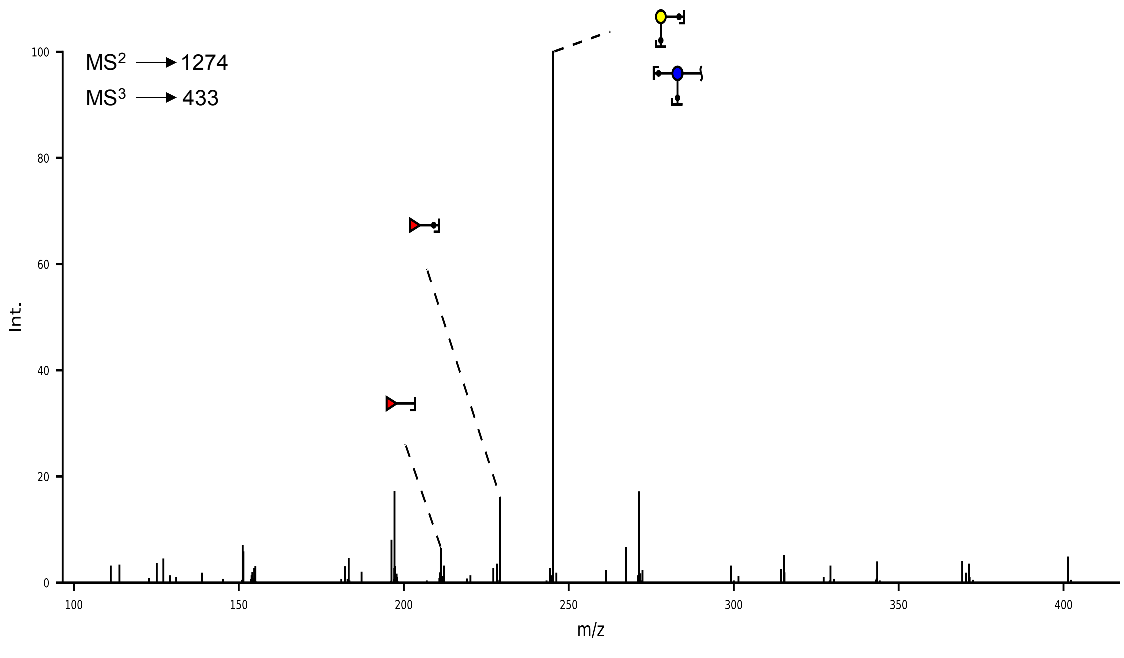

(c)

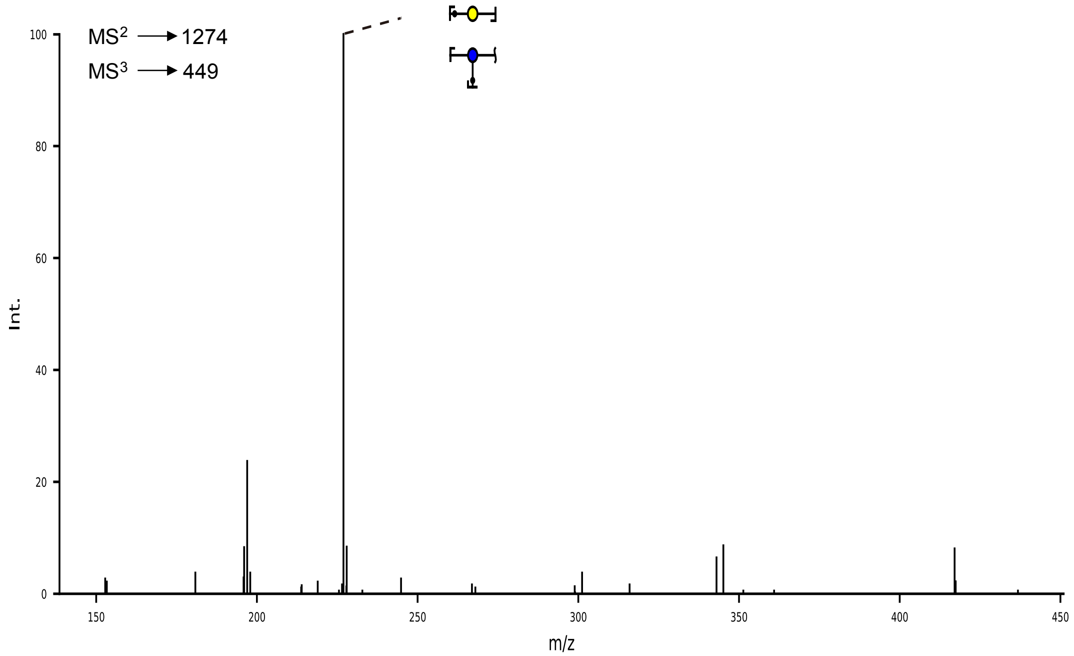
MS2 → 1274
MS3 → 449
100
80
60
40
20
0
Int.
150
200
250
300
350
400
450
m/z

(d)

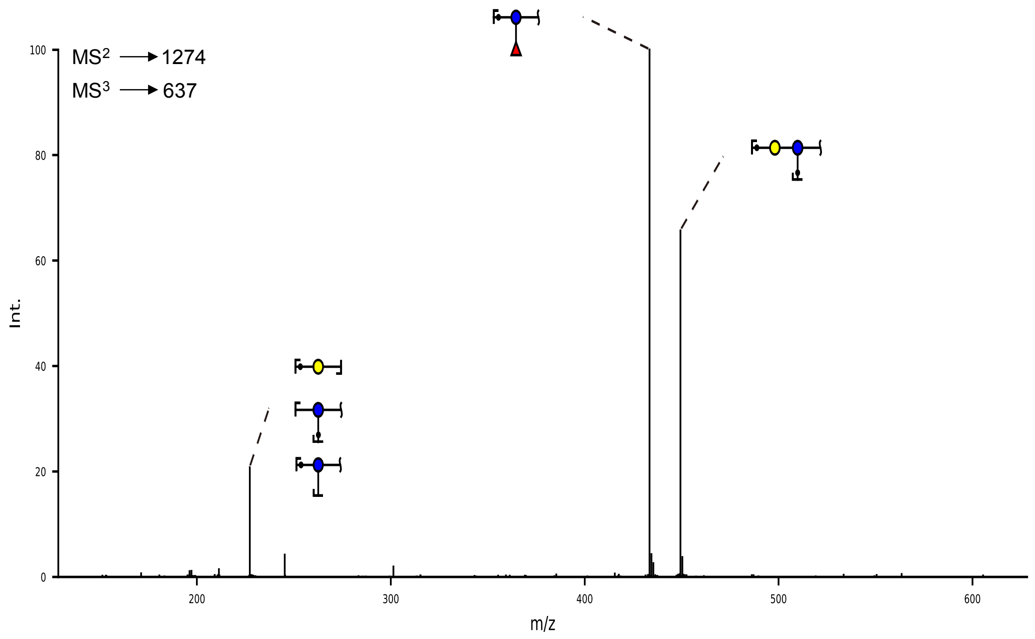

(e)

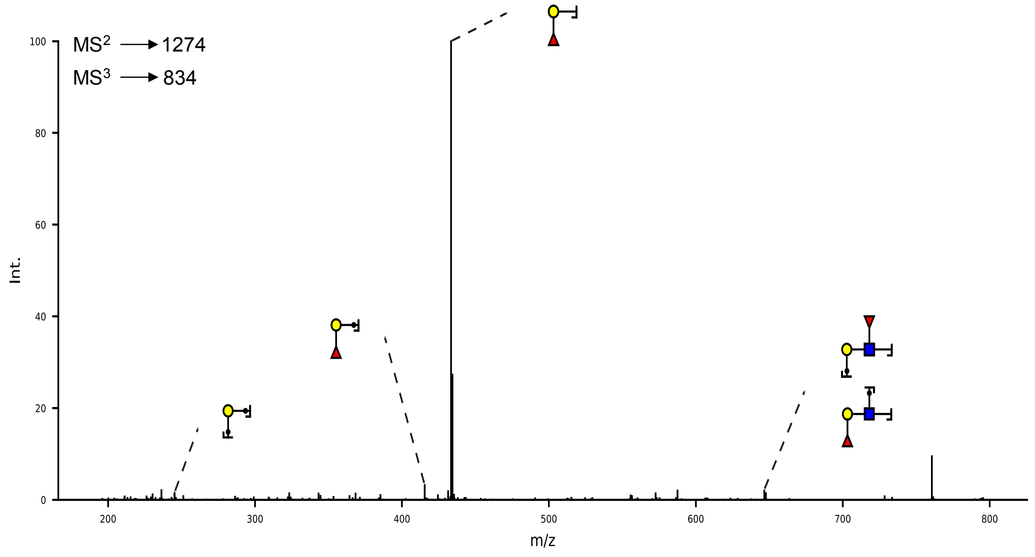

(f)

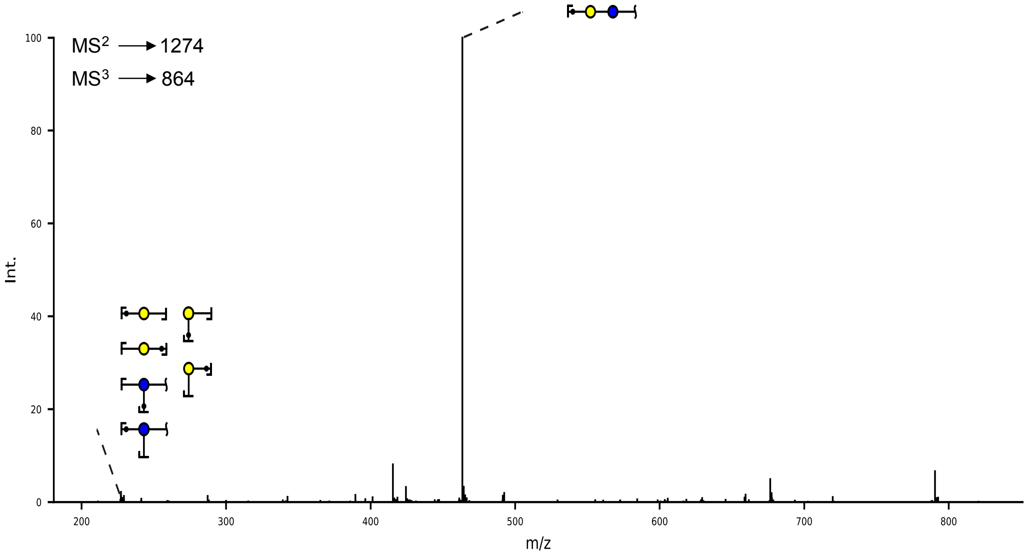

MS2 → 1274
MS3 → 864
Int.
m/z
0
20
40
60
80
100
200
300
400
500
600
700
800

(g)

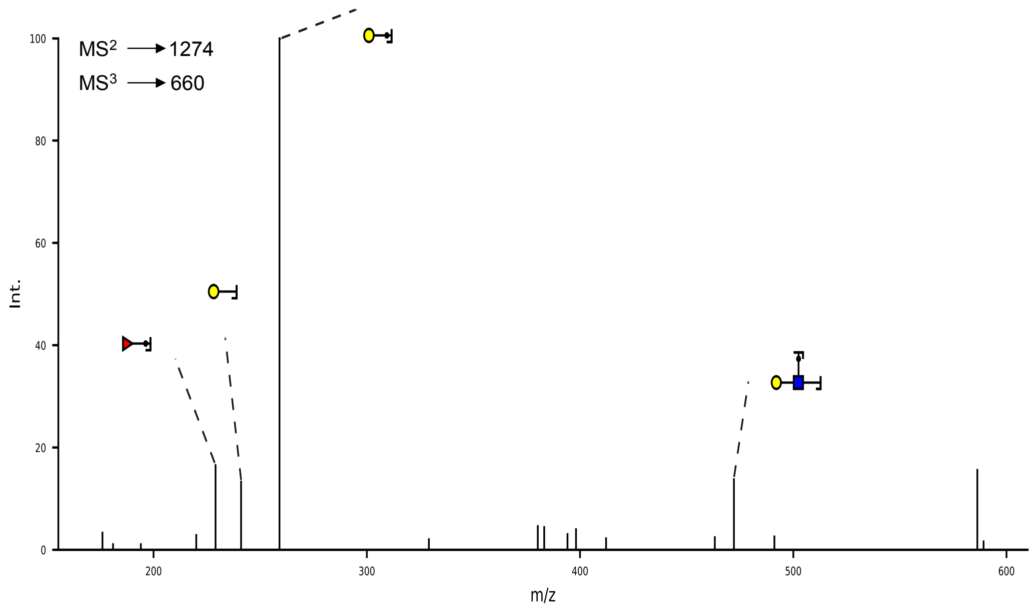

Figure S2. Produced MS^n spectra with peak annotation for the sample mixture, LNDFH-I and LNnDFH-II with a molar ratio 1:1, using GIPS-mix. (a), MS^2 spectrum of m/z 1274, (b) MS^3 spectrum m/z 433, (c) MS^3 spectrum m/z 449, (d) MS^3 spectrum m/z 637, (e) MS^3 spectrum m/z 834, (f) MS^3 spectrum m/z 864, (g) MS^3 spectrum m/z 660.

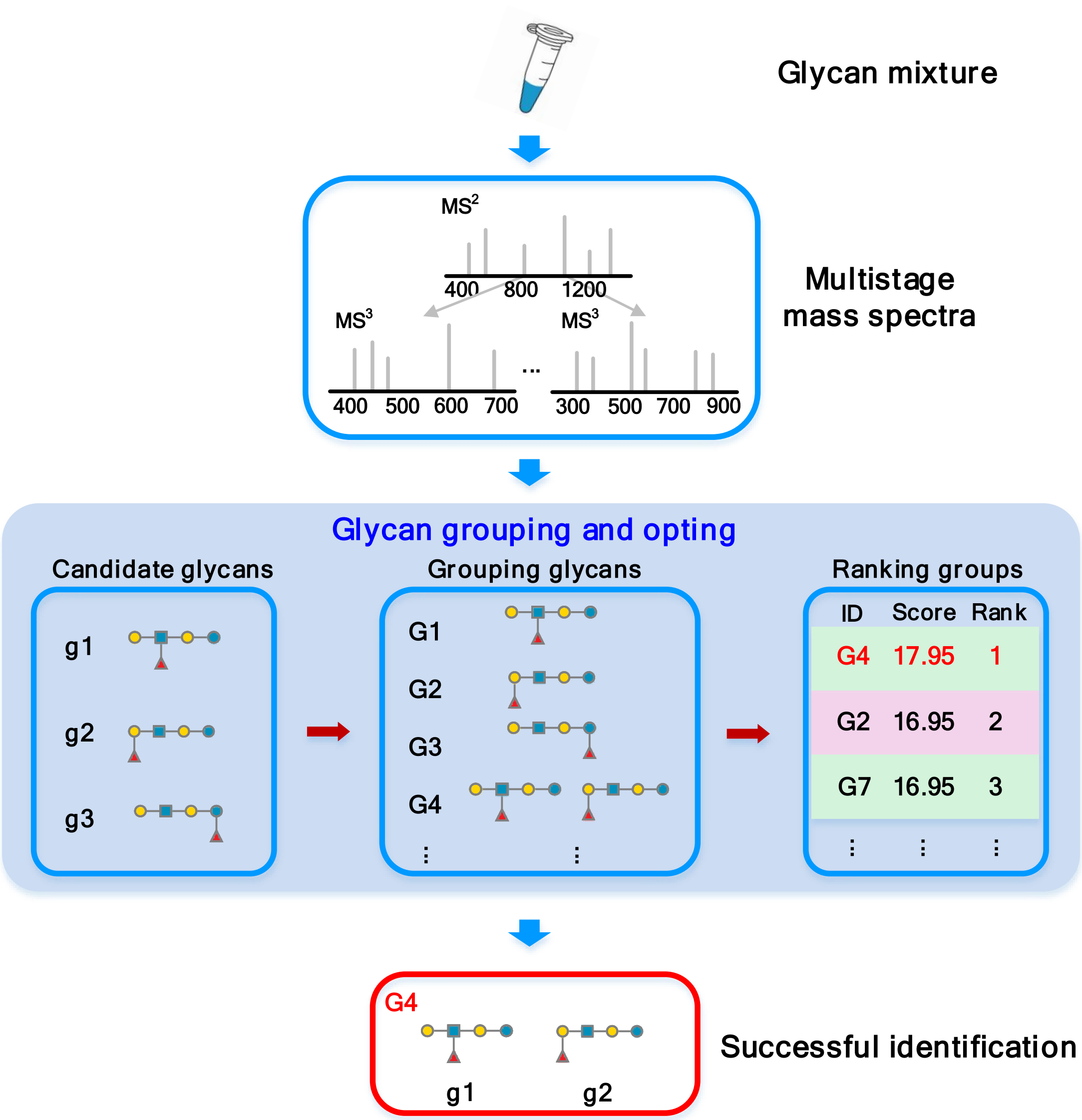

Figure S3. Identification by GIPS-mix of the components in a mixture of LNFP-I and LNFP-II with a molar ratio of 1:1.

The MS^1 spectrum of the mixture gave a permethylated MNa$^+$ at *m/z* 1100, indicating that the glycan components have a molecular mass of 853 *Da.* From the glycan structure database GlyTouCan, we identified 13 candidate glycans with this molecular mass, showing 3 branching structures, and for each branching pattern, we select a glycan as its representative, denoted as g_1, g_2,g_3. Here, g_1 denotes the branching pattern of LNFP-II, and g_2 denotes that of LNFP-I. The *glycan grouping and opting* module enumerated all 7 groupings of these candidate glycans, i.e., $G_1 = \{g_1\}$, $G_2 = \{g_2\}$, $G_3 = \{g_3\}$, $G_4 = \{g_1,g_2\}$, $G_5 = \{g_1,g_3\}$, $G_6 = \{g_2,g_3\}$, $G_7 = \{g_1, g_2, g_3\}$. Among these groups, G_4 showed the highest similarity (17.95) between their theoretical spectra and the experimental spectra of the mixture.

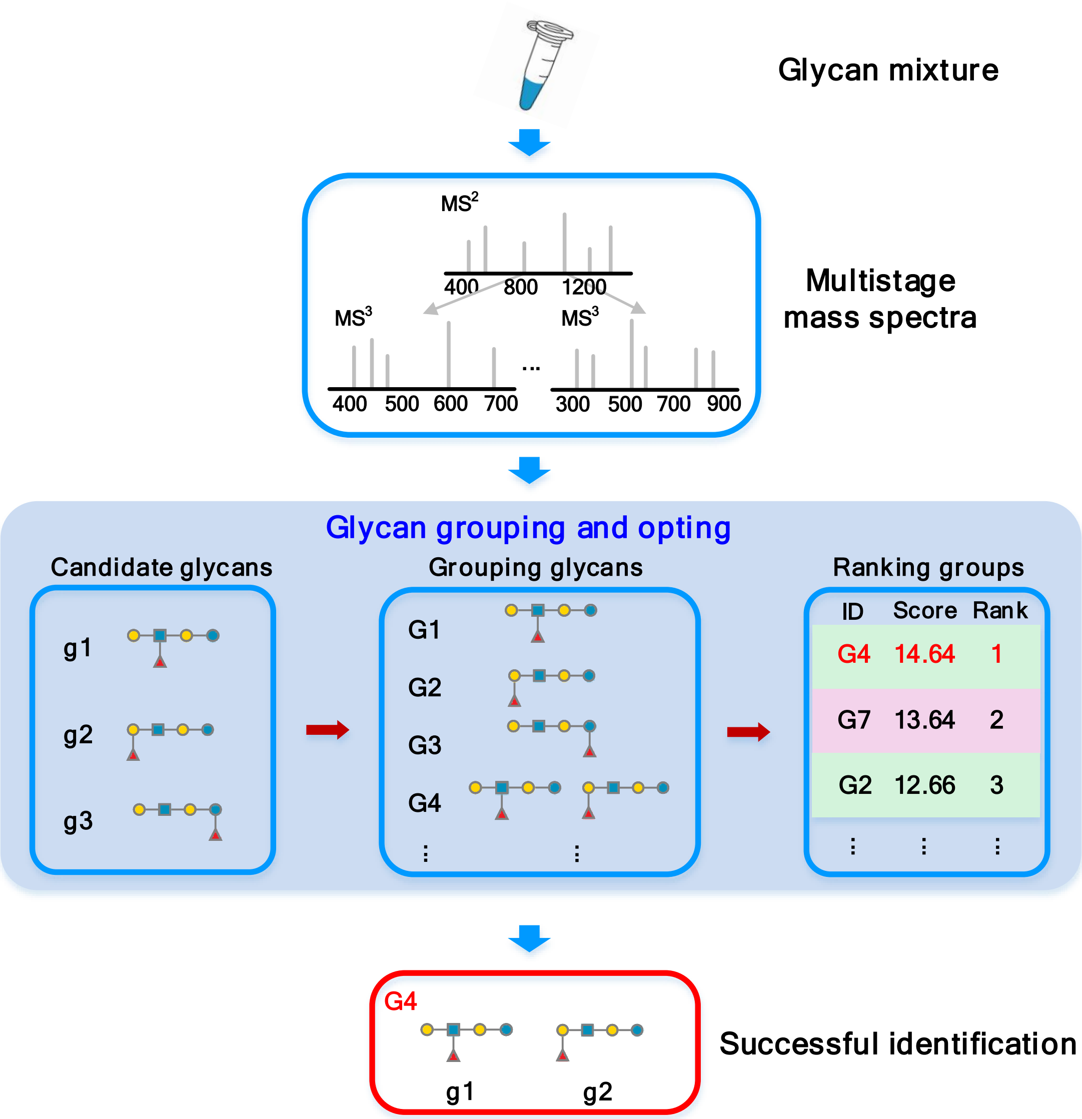

Figure S4. Identification by GIPS-mix of the components in a mixture of LNFP-I and LNFP-II with a molar ratio of 1:3.

The MS^1 spectrum of the mixture gave a permethylated MNa^+ at *m/z* 1100, indicating that the glycan components have a molecular mass of 853 *Da.* From the glycan structure database GlyTouCan, we identified 13 candidate glycans with this molecular mass, showing 3 branching structures, and for each branching pattern, we select a glycan as its representative, denoted as g_1, g_2,g_3. Here, g_1 denotes the branching pattern of LNFP-II, and g_2 denotes that of LNFP-I. The *glycan grouping and opting* module enumerated all 7 groupings of these candidate glycans, i.e., $G_1 = \{g_1\}$, $G_2 = \{g_2\}$, $G_3 = \{g_3\}$, $G_4 = \{g_1,g_2\}$, $G_5 = \{g_1,g_3\}$, $G_6 = \{g_2,g_3\}$, $G_7 = \{g_1, g_2, g_3\}$. Among these groups, G_4 showed the highest similarity (14.64) between their theoretical spectra and the experimental spectra of the mixture.

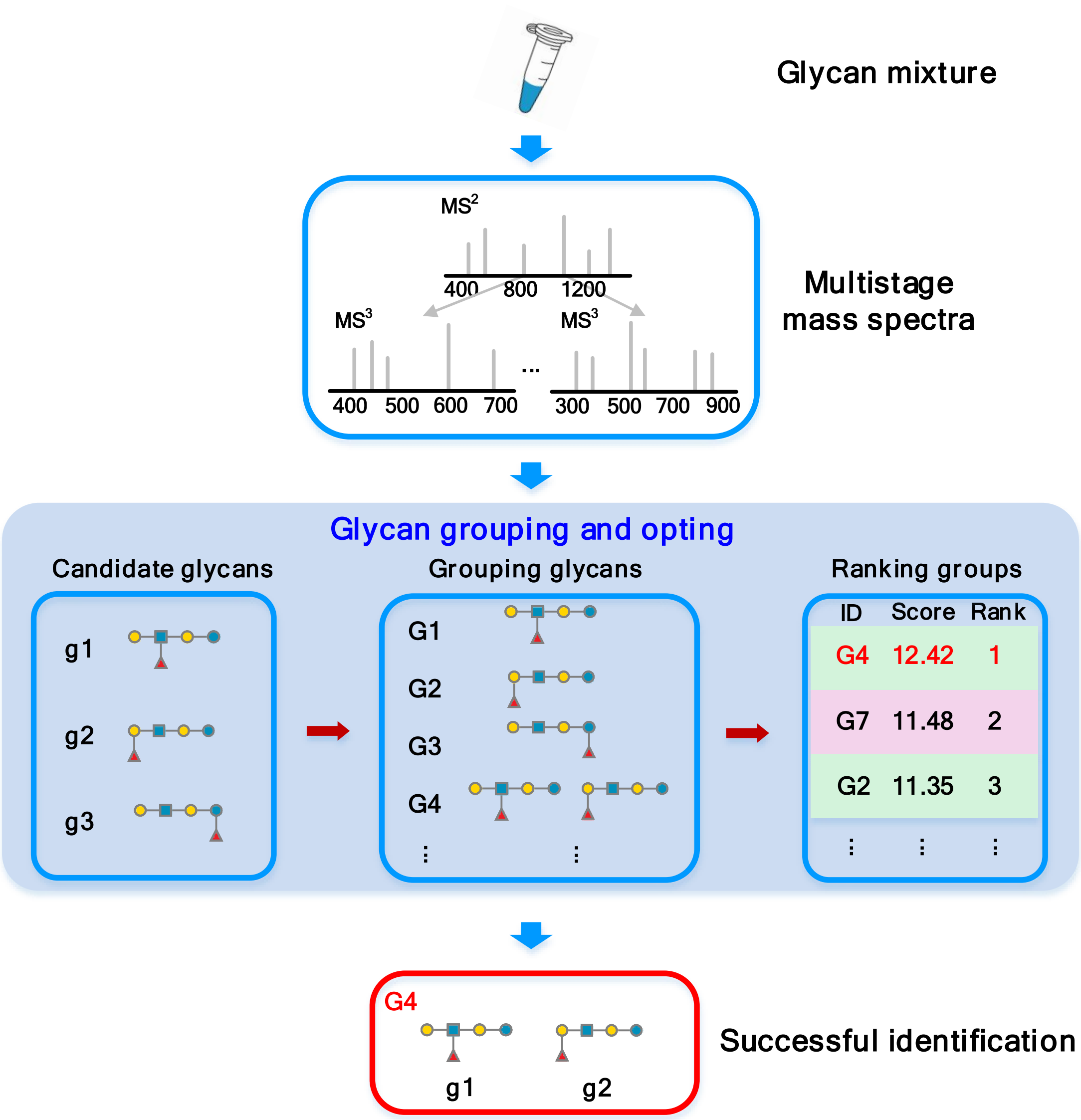

Figure S5. Identification by GIPS-mix of the components in a mixture of LNnFP-I and LNFP-II with a molar ratio of 1:1.

The MS^1 spectrum of the mixture gave a permethylated MNa^+ at *m/z* 1100, indicating that the glycan components have a molecular mass of 853 *Da*. From the glycan structure database GlyTouCan, we identified 13 candidate glycans with this molecular mass, showing 3 branching structures, and for each branching pattern, we select a glycan as its representative, denoted as g_1, g_2,g_3. Here, g_1 denotes the branching pattern of LNFP-II, and g_2 denotes that of LNnFP-I. The *glycan grouping and opting* module enumerated all 7 groupings of these candidate glycans, i.e., $G_1 = \{g_1\}$, $G_2 = \{g_2\}$, $G_3 = \{g_3\}$, $G_4 = \{g_1,g_2\}$, $G_5 = \{g_1,g_3\}$, $G_6 = \{g_2,g_3\}$, $G_7 = \{g_1, g_2, g_3\}$. Among these groups, G_4 showed the highest similarity (12.42) between their theoretical spectra and the experimental spectra of the mixture.

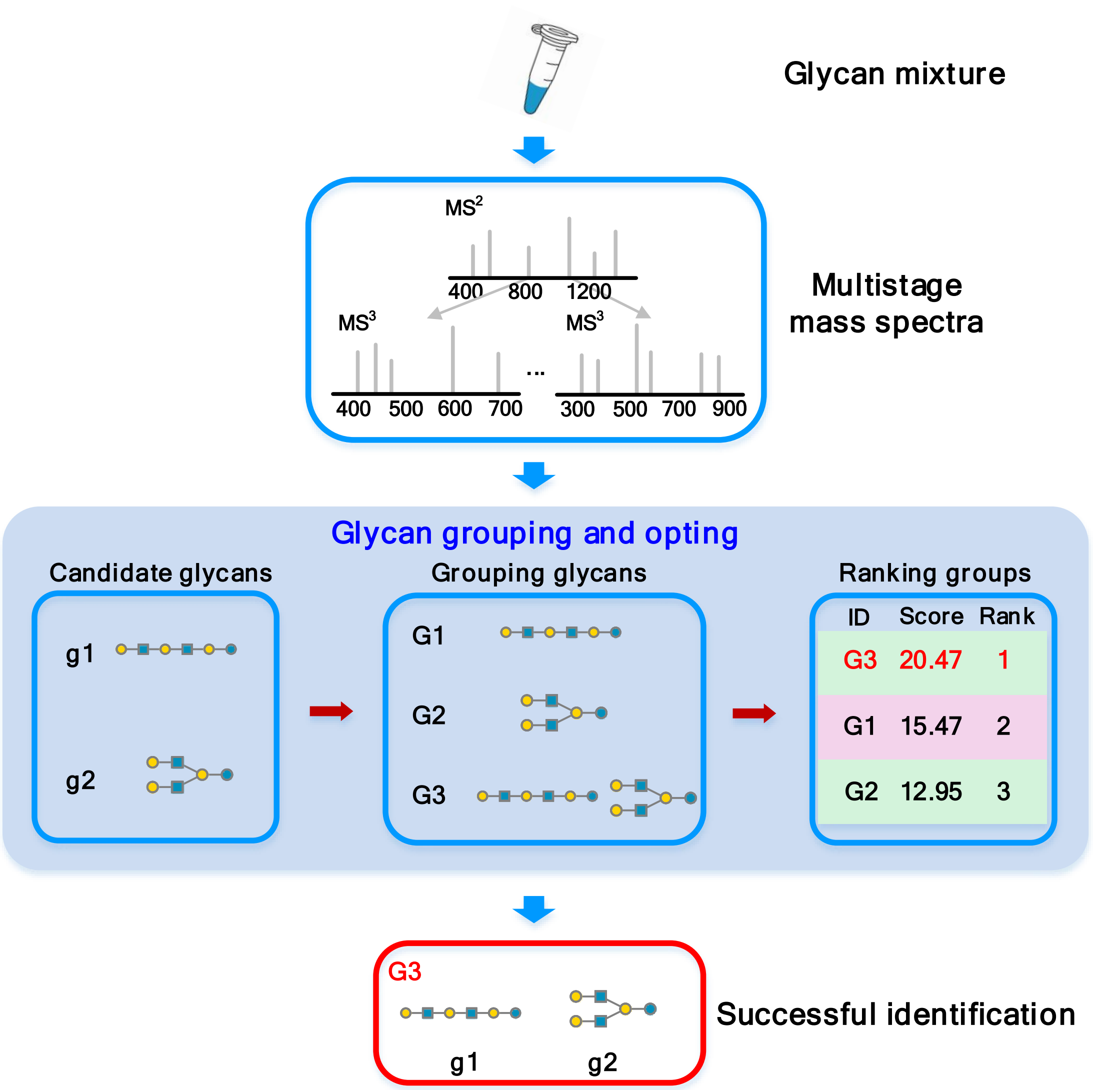

Figure S6. Identification by GIPS-mix of the components in a mixture of LNH and pLNH with a molar ratio of 1:1.

The MS^1 spectrum of the mixture gave a permethylated MNa^+ at *m/z*1375, indicating that the glycan components have a molecular mass of 1072 *Da.* From the glycan structure database GlyTouCan, we identified 10 candidate glycans with this molecular mass, showing 2 branching structures, and for each branching pattern, we select a glycan as its representative, denoted as g_1, g_2. Here, g_1 denotes the branching pattern of pLNH, and g_2 denotes that of LNH. The *glycan grouping and opting* module enumerated all 3 groupings of these candidate glycans, i.e., $G_1 = \{g_1\}$, $G_2 = \{g_2\}$, $G_3 = \{g_1,g_2\}$. Among these groups, G_3 showed the highest similarity (20.47) between their theoretical spectra and the experimental spectra of the mixture.

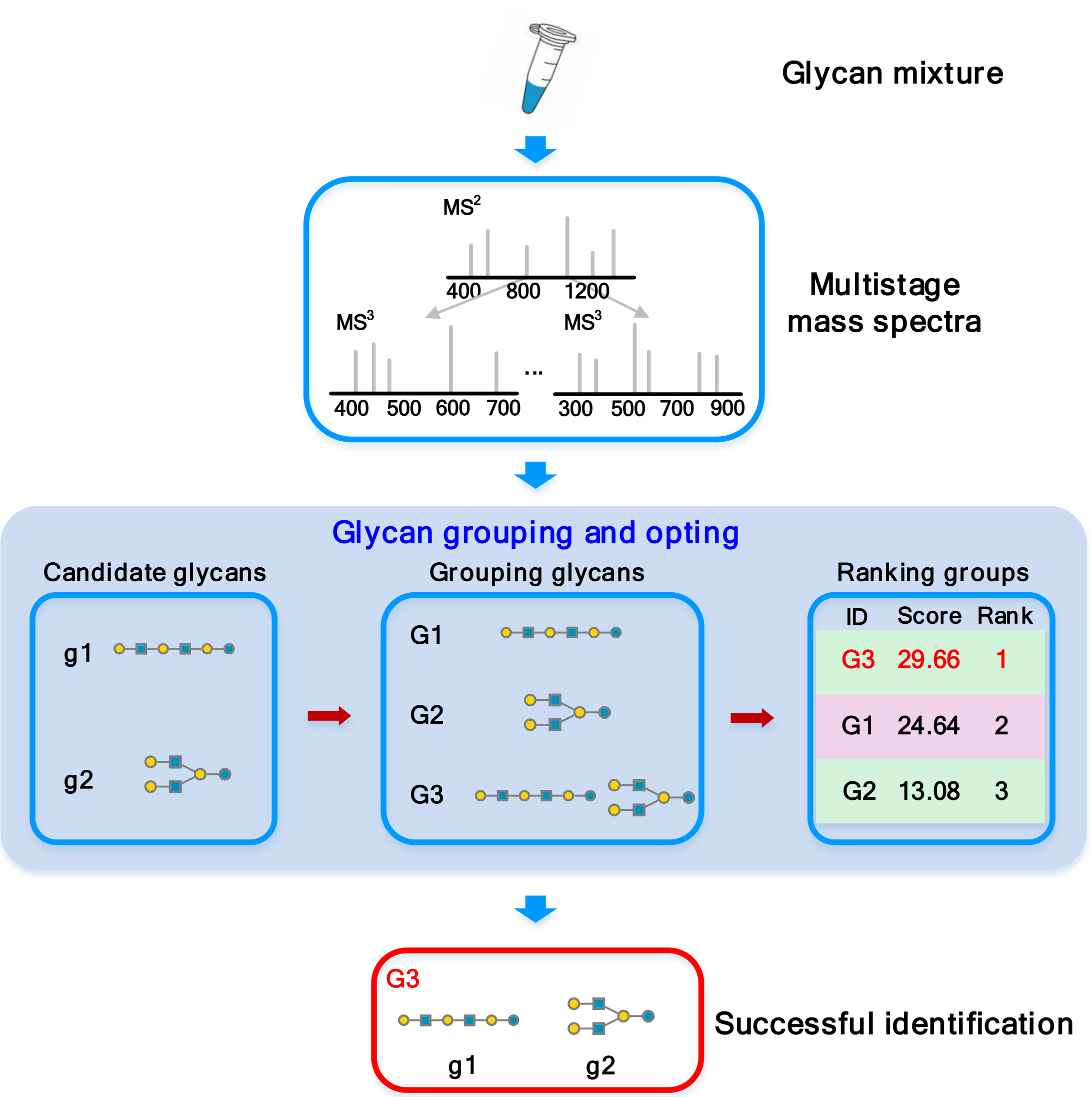

Figure S7. Identification by GIPS-mix of the components in a mixture of LNH and pLNH with a molar ratio of 1:3.

The MS^1 spectrum of the mixture gave a permethylated MNa^+ at *m/z*1375, indicating that the glycan components have a molecular mass of 1072 *Da.* From the glycan structure database GlyTouCan, we identified 10 candidate glycans with this molecular mass, showing 2 branching structures, and for each branching pattern, we select a glycan as its representative, denoted as g_1, g_2. Here, g_1 denotes the branching pattern of pLNH, and g_2 denotes that of LNH. The *glycan grouping and opting* module enumerated all 3 groupings of these candidate glycans, i.e., $G_1 = \{g_1\}$, $G_2 = \{g_2\}$, $G_3 = \{g_1,g_2\}$. Among these groups, G_3 showed the highest similarity (29.66) between their theoretical spectra and the experimental spectra of the mixture.

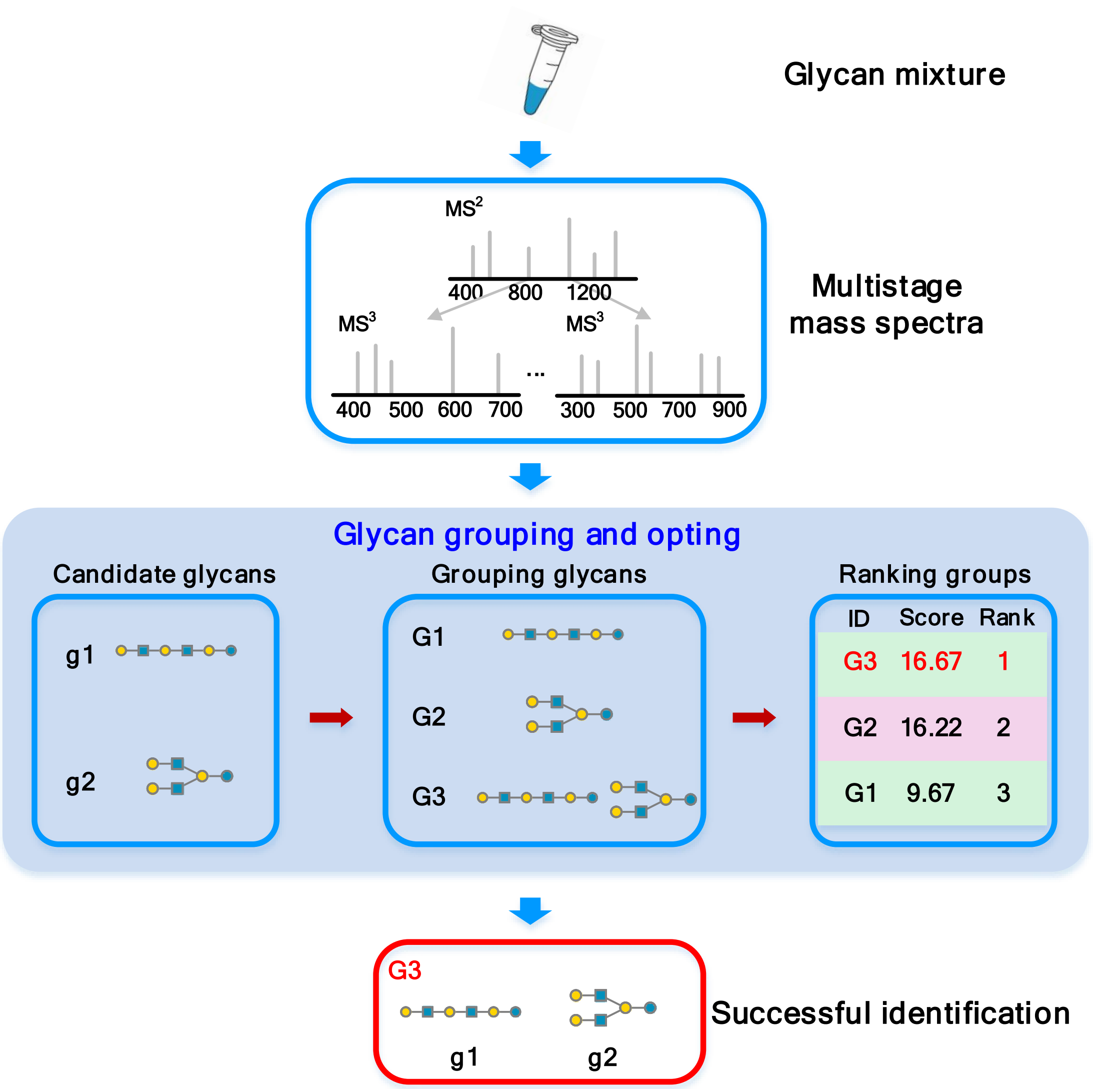

Figure S8. Identification by GIPS-mix of the components in a mixture of LNH and pLNH with a molar ratio of 3:1.

The MS[1] spectrum of the mixture gave a permethylated MNa^+ at *m/z*1375, indicating that the glycan components have a molecular mass of 1072 *Da*. From the glycan structure database GlyTouCan, we identified 10 candidate glycans with this molecular mass, showing 2 branching structures, and for each branching pattern, we select a glycan as its representative, denoted as g_1, g_2. Here, g_1 denotes the branching pattern of pLNH, and g_2 denotes that of LNH. The *glycan grouping and opting* module enumerated all 3 groupings of these candidate glycans, i.e., $G_1 = \{g_1\}$, $G_2 = \{g_2\}$, $G_3 = \{g_1,g_2\}$. Among these groups, G_3 showed the highest similarity (16.67) between their theoretical spectra and the experimental spectra of the mixture.

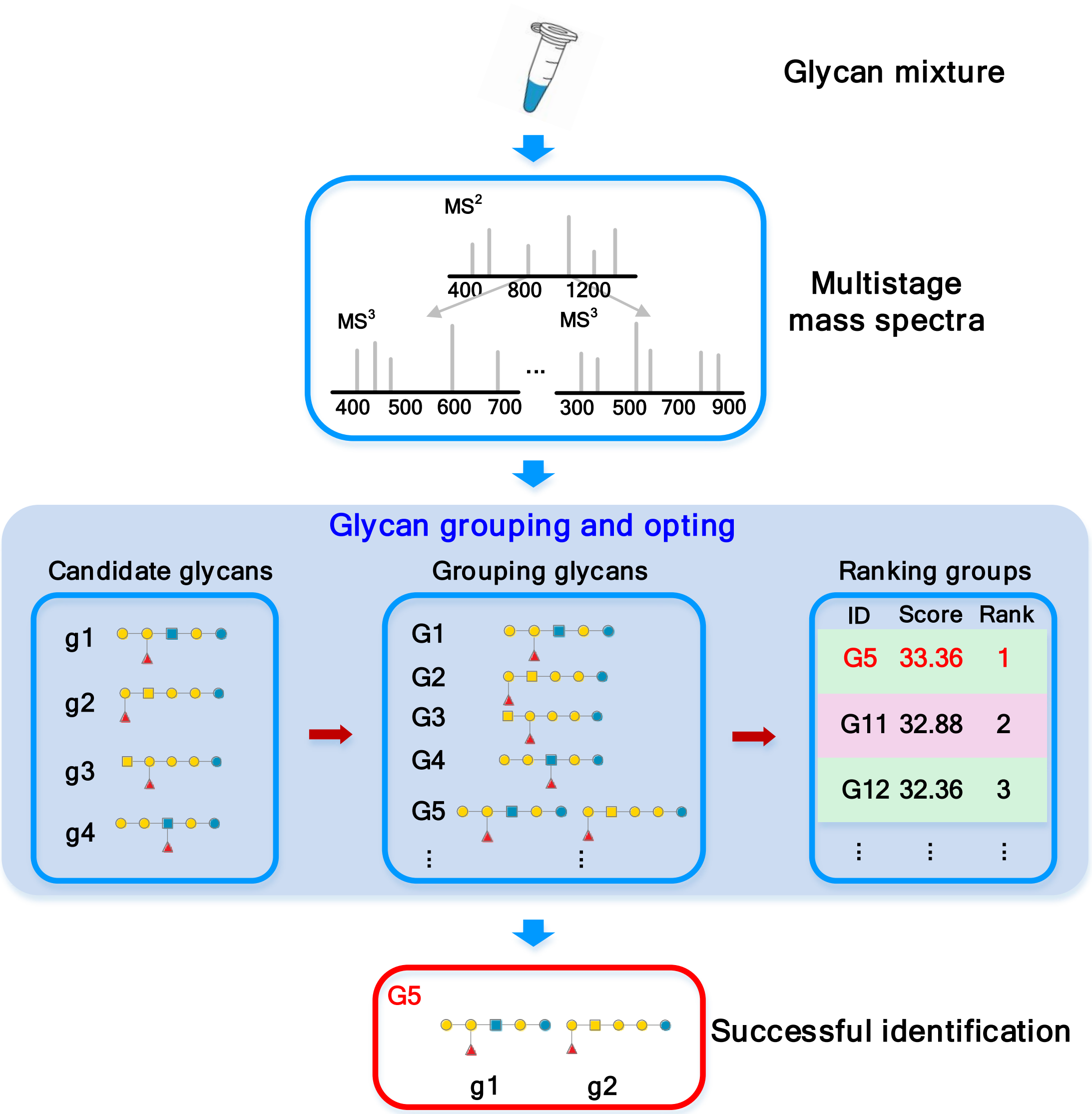

Figure S9. Identification by GIPS-mix of the components in a mixture of B-Tetra-T2 and Globo-H-Hexa with a molar ratio of 1:1.

The MS^1 spectrum of the mixture gave a permethylated MNa^+ at *m/z*1304, indicating that the glycan components have a molecular mass of 1015 *Da*. From the glycan structure database GlyTouCan, we identified 12 candidate glycans with this molecular mass, showing 4 branching structures, and for each branching pattern, we select a glycan as its representative, denoted as g_1, g_2,g_3,g_4. Here, g_1 denotes the branching pattern of B-Tetra-T2, and g_2 denotes that of Globo-H-Hexa. The *glycan grouping and opting* module enumerated all 4 groupings of these candidate glycans, i.e., $G_1 = \{g_1\}$, $G_2 = \{g_2\}$, $G_3 = \{g_3\}$, $G_4 = \{g_4\}$, $G_5 = \{g_1,g_2,\}$, $G_6 = \{g_1,g_3\}$, $G_7 = \{g_1,g_4\}$, $G_8 = \{g_2,g_3\}$, $G_9 = \{g_2,g_4\}$, $G_{10} = \{g_3,g_4\}$, $G_{11} = \{g_1,g_2,g_3\}$, $G_{12} = \{g_1,g_2,g_4\}$, $G_{13} = \{g_1,g_3,g_4\}$, $G_{14} = \{g_2,g_3,g_4\}$, $G_{15} = \{g_1,g_2,g_3,g_4\}$.Among these groups, G_5 showed the highest similarity (33.46) between their theoretical spectra and the experimental spectra of the mixture.

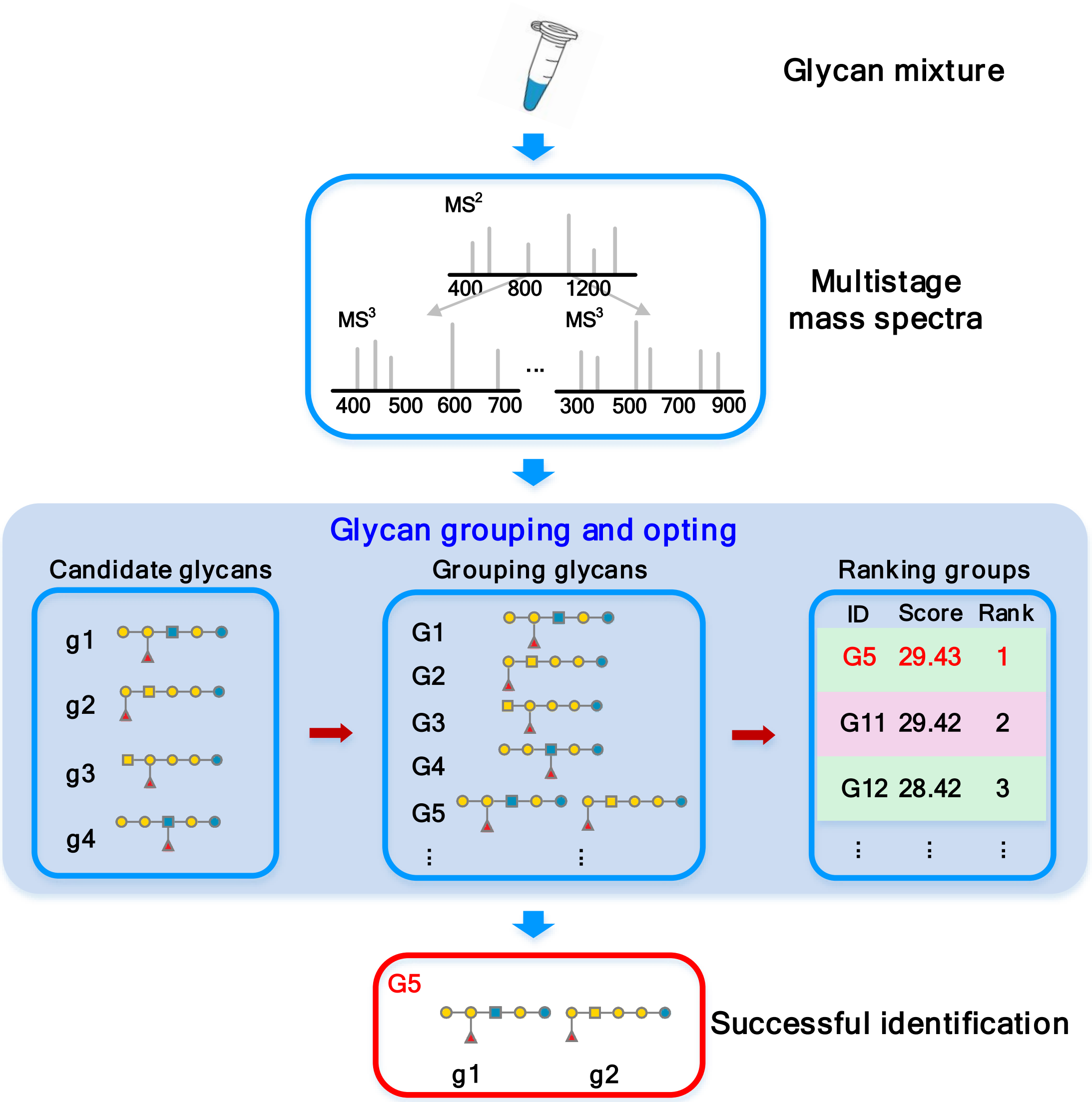

Figure S10. Identification by GIPS-mix of the components in a mixture of B-Tetra-T2 and Globo-H-Hexa with a molar ratio of 1:3.

The MS[1] spectrum of the mixture gave a permethylated MNa+ at *m/z*1304, indicating that the glycan components have a molecular mass of 1015 *Da*. From the glycan structure database GlyTouCan, we identified 12 candidate glycans with this molecular mass, showing 4 branching structures, and for each branching pattern, we select a glycan as its representative, denoted as g_1, g_2,g_3,g_4. Here, g_1 denotes the branching pattern of B-Tetra-T2, and g_2 denotes that of Globo-H-Hexa. The *glycan grouping and opting* module enumerated all 4 groupings of these candidate glycans, i.e., $G_1 = \{g_1\}$, $G_2 = \{g_2\}$, $G_3 = \{g_3\}$, $G_4 = \{g_4\}$, $G_5 = \{g_1,g_2,\}$, $G_6 = \{g_1,g_3\}$, $G_7 = \{g_1,g_4\}$, $G_8 = \{g_2,g_3\}$, $G_9 = \{g_2,g_4\}$, $G_{10} = \{g_3,g_4\}$, $G_{11} = \{g_1,g_2,g_3\}$, $G_{12} = \{g_1,g_2,g_4\}$, $G_{13} = \{g_1,g_3,g_4\}$, $G_{14} = \{g_2,g_3,g_4\}$, $G_{15} = \{g_1,g_2,g_3,g_4\}$.Among these groups, G_5 showed the highest similarity (29.43) between their theoretical spectra and the experimental spectra of the mixture.

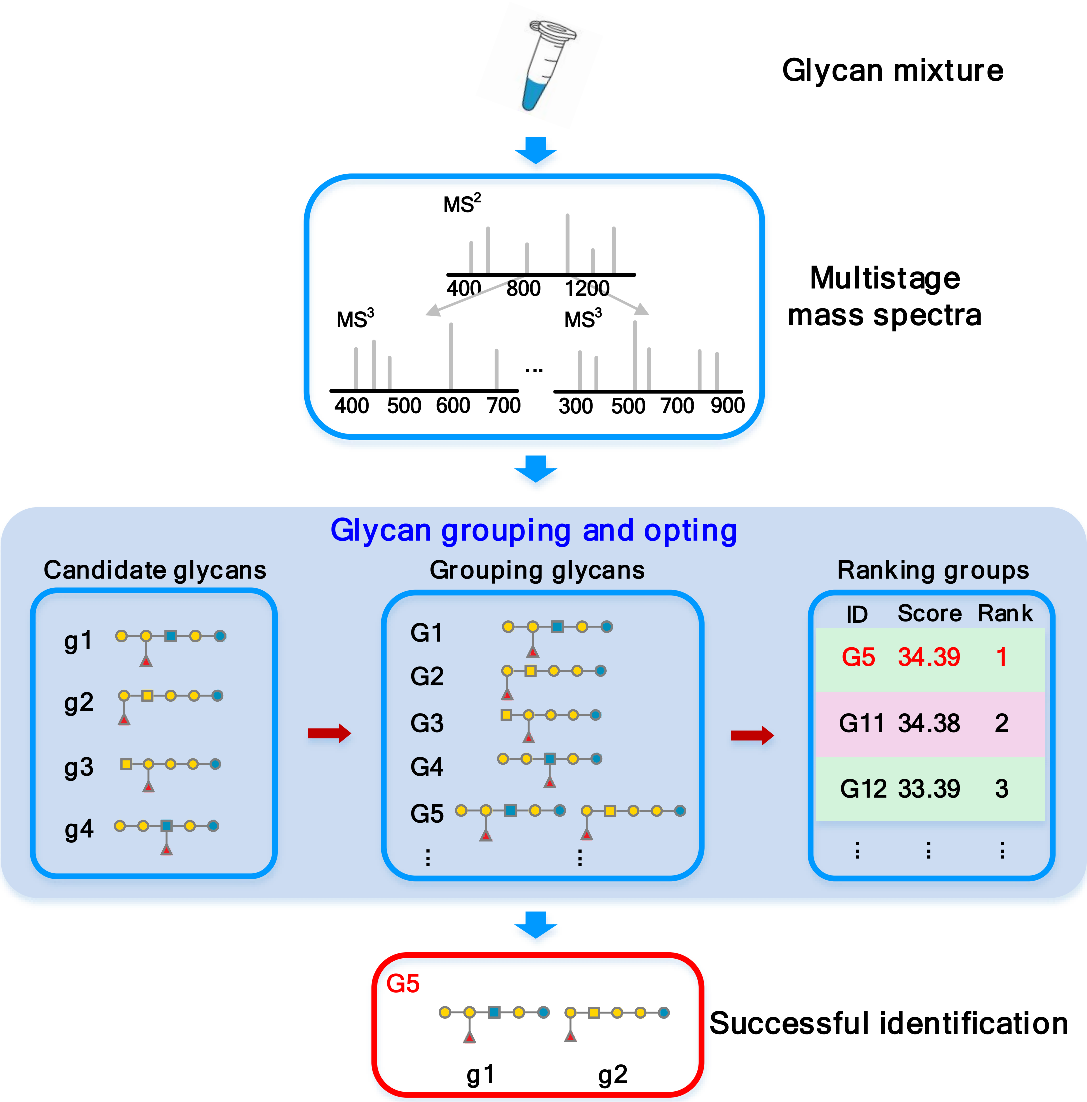

Figure S11. Identification by GIPS-mix of the components in a mixture of B-Tetra-T2 and Globo-H-Hexa with a molar ratio of 3:1.

The MS[1] spectrum of the mixture gave a permethylated MNa+ at *m/z*1304, indicating that the glycan components have a molecular mass of 1015 *Da.* From the glycan structure database GlyTouCan, we identified 12 candidate glycans with this molecular mass, showing 4 branching structures, and for each branching pattern, we select a glycan as its representative, denoted as g_1, g_2,g_3,g_4. Here, g_1 denotes the branching pattern of B-Tetra-T2, and g_2 denotes that of Globo-H-Hexa. The *glycan grouping and opting* module enumerated all 4 groupings of these candidate glycans, i.e., $G_1 = \{g_1\}$, $G_2 = \{g_2\}$, $G_3 = \{g_3\}$, $G_4 = \{g_4\}$, $G_5 = \{g_1,g_2,\}$, $G_6 = \{g_1,g_3\}$, $G_7 = \{g_1,g_4\}$, $G_8 = \{g_2,g_3\}$, $G_9 = \{g_2,g_4\}$, $G_{10} = \{g_3,g_4\}$, $G_{11} = \{g_1,g_2,g_3\}$, $G_{12} = \{g_1,g_2,g_4\}$, $G_{13} = \{g_1,g_3,g_4\}$, $G_{14} = \{g_2,g_3,g_4\}$, $G_{15} = \{g_1,g_2,g_3,g_4\}$.Among these groups, G_5 showed the highest similarity (34.39) between their theoretical spectra and the experimental spectra of the mixture.

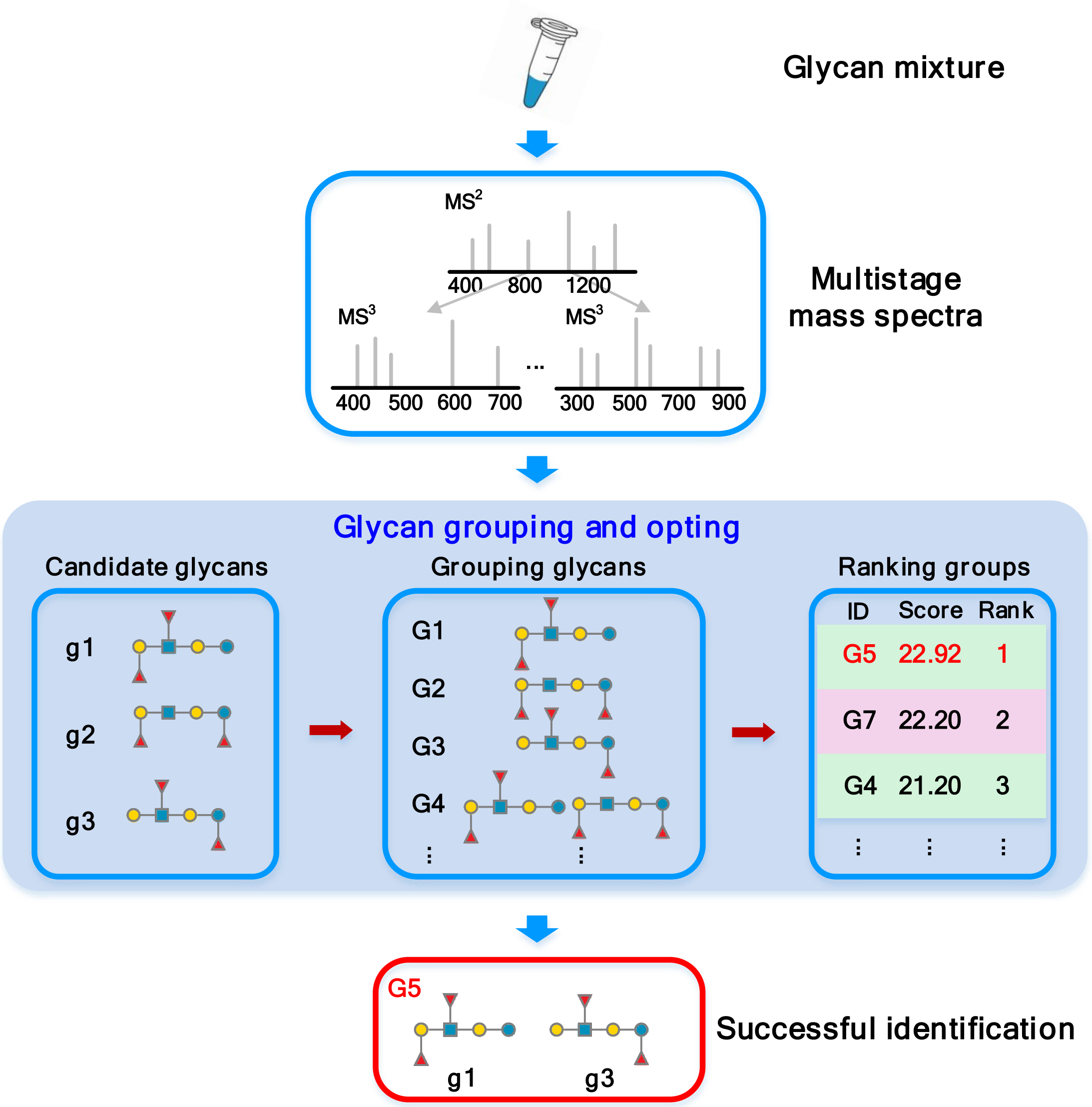

Figure S12. Identification by GIPS-mix of the components in a mixture of LNDFH-I and LNnDFH-II with a molar ratio of 1:1.

The MS[1] spectrum of the mixture gave a permethylated MNa[+] at *m/z* 1274, indicating that the glycan components have a molecular mass of 999 *Da.* From the glycan structure database GlyTouCan, we identified 8 candidate glycans with this molecular mass, showing 3 branching structures, and for each branching pattern, we select a glycan as its representative, denoted as g_1, g_2,g_3. Here, g_1 denotes the branching pattern of LNDFH-I, and g_3 denotes that of LNnDFH-II. The *glycan grouping and opting* module enumerated all 7 groupings of these candidate glycans, i.e., $G_1 = \{g_1\}$, $G_2 = \{g_2\}$, $G_3 = \{g_3\}$, $G_4 = \{g_1,g_2\}$, $G_5 = \{g_1,g_3\}$, $G_6 = \{g_2,g_3\}$, $G_7 = \{g_1, g_2, g_3\}$. Among these groups, G_5 showed the highest similarity (22.92) between their theoretical spectra and the experimental spectra of the mixture.

(a)

(b)

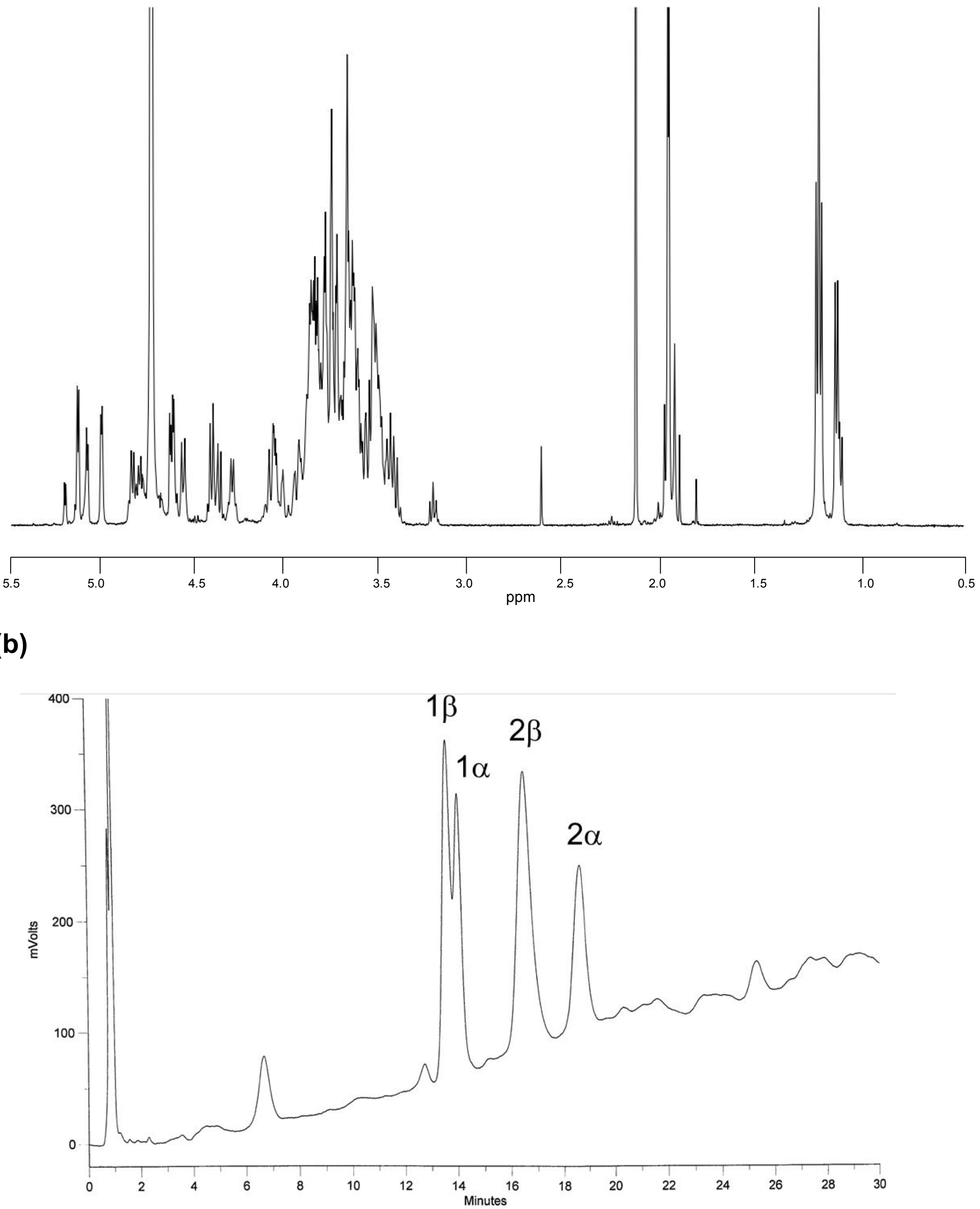

Figure S13. 1H-NMR spectrum (a) and PGC-HPLC profile (b) of HMO fraction DP9. Peaks α and β were produced by different configurations of one oligosaccharide referring to the anomeric C1 atom.

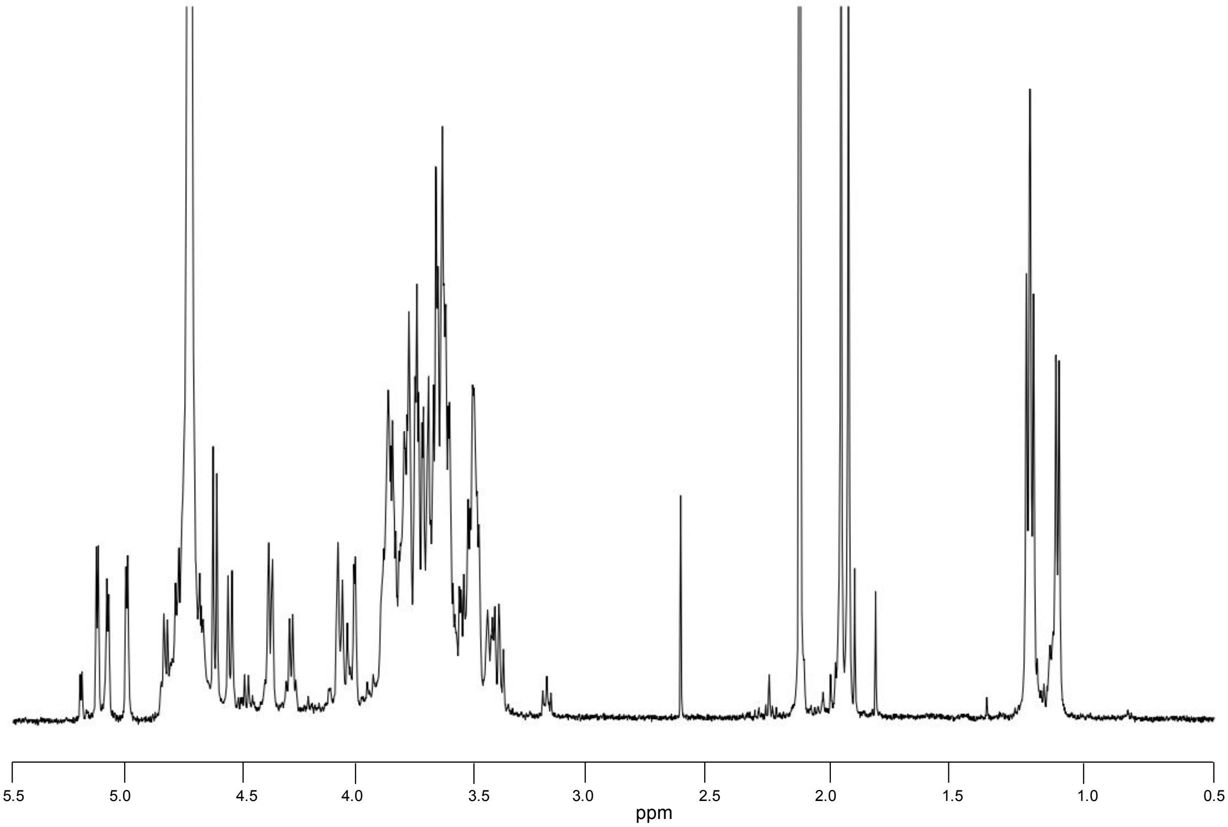

Figure S14. ^{1}H-NMR spectrum of oligosaccharide TFpLNH.

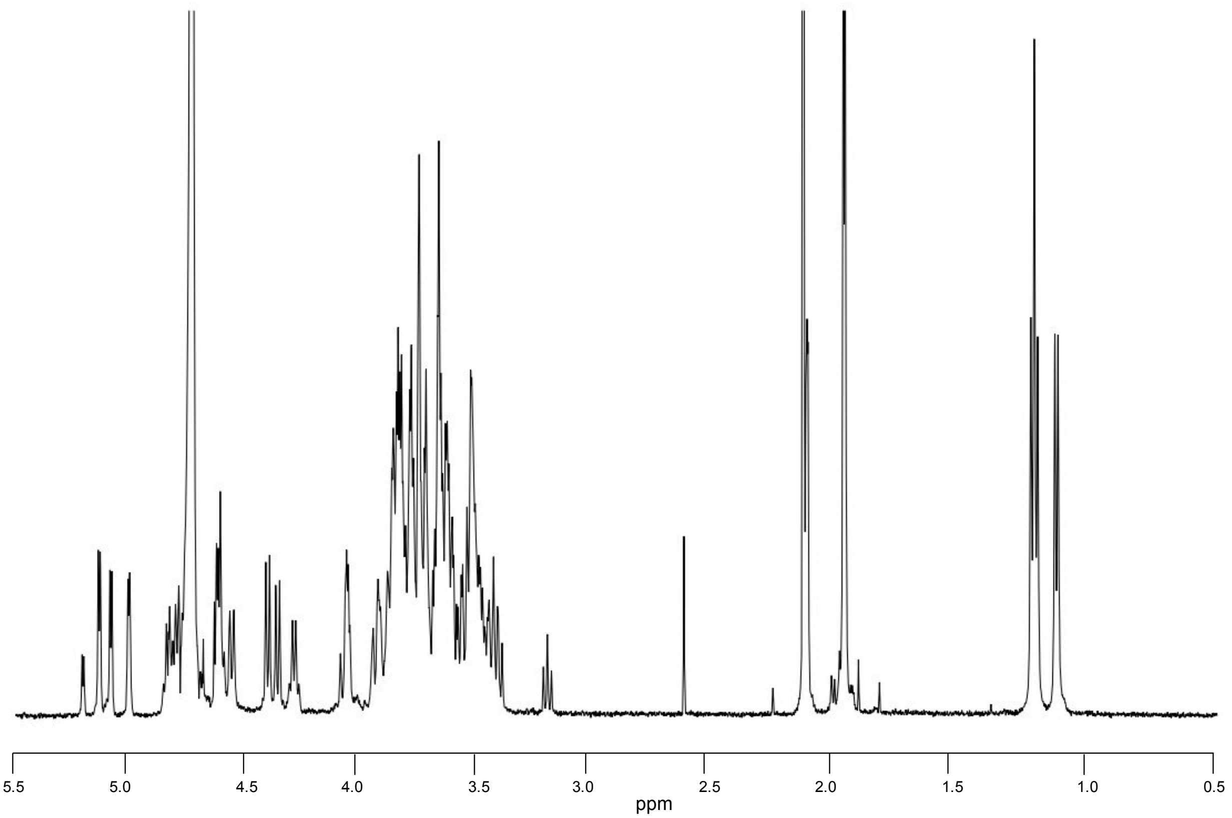

Figure S15. ^{1}H-NMR spectrum of oligosaccharide TFLNH.

Supplementary References

S1. Huang, C.; Sun, S.; Yan, J.; Wang, H.; Zhou, J.; Gao, H.; Xie, W.; Li, Y.; Chai, W. Identification of Carbohydrate Peripheral Epitopes Important for Recognition by Positive-ion MALDI Multistage Mass Spectrometry. *Carbohyd. Polym.* **2020**, *229*, 115528.

S2. Choo, M.; Tan, H. L.; Ding, V.; Castangia, R.; Belgacem, O.; Liau, B.; Hartley-Tassell, L.; Haslam, S. M.; Dell, A.; Choo, A. Characterization of H Type 1 and Type 1 N-acetyllactosamine Glycan Epitopes on Ovarian Cancer Specifically Recognized by the Anti-glycan Monoclonal Antibody mAb-A4. *J. Biol. Chem.* **2017**, *292*, 6163–6176.

S3. Ashline, D. J.; Hanneman, A. J. S.; Zhang, H.; Reinhold, V. N. Structural Documentation of Glycan Epitopes: Sequential Mass Spectrometry and Spectral Matching. *J. Am. Soc. Spectrom.* **2014**, *25*, 444–453.

S4. Sun, S.; Huang, C.; Wang, Y.; Liu, Y.; Zhang, J.; Zhou, J.; Gao, F.; Yang, F.; Chen, R.; Mulloy, B.; Chai, W.; Li, Y.; Bu, D. Toward Automated Identification of Glycan Branching Patterns Using Multistage Mass Spectrometry with Intelligent Precursor Selection. *Anal. Chem*. **2018**, *90*, 14412–14422.

S5. Ashline, D. J.; Yu, Y.; Lasanajak, Y.; Song, X.; Hu, L.; Ramani, S.; Prasad, V.; Estes, M. K.; Cummings, R. D.; Smith, S. F.; Reinhold, V. N. Structural Characterization by Multistage Mass Spectrometry (MSn) of Human Milk Glycans Recognized by Human Rotaviruses. *Mol. Cell. Proteomics* **2014**, *13*, 2961–2974.
